# Identification of candidate genes related to calanolide biosynthesis by transcriptome sequencing of *Calophyllum brasiliense* (Calophyllaceae)

**DOI:** 10.1186/s12870-016-0862-9

**Published:** 2016-08-15

**Authors:** Hilda-Beatriz Gómez-Robledo, Francisco Cruz-Sosa, Antonio Bernabé-Antonio, Antonio Guerrero-Analco, José Luis Olivares-Romero, Alexandro Alonso-Sánchez, Emanuel Villafán, Enrique Ibarra-Laclette

**Affiliations:** 1 Facultad de Ciencias de la Salud, Universidad Anáhuac, 52786 Estado de México, México; 2 Laboratorio de Bioquímica y Biología Molecular, Escuela Médico Militar, Universidad del Ejército y Fuerza Aérea, 11200 Ciudad de México, México; 3 Departamento de Biotecnología, Universidad Autónoma Metropolitana Unidad Iztapalapa (UAM-Iztapalapa), 09340 Ciudad de México, México; 4 Departamento de Madera, Celulosa y Papel, Centro Universitario de Ciencias Exactas e Ingenierías, Universidad de Guadalajara, 45100 Zapopan, Jalisco México; 5 Red de Estudios Moleculares Avanzados, Instituto de Ecología A.C, 91070 Xalapa, Veracruz México

## Abstract

**Background:**

*Calophyllum brasiliense* is highlighted as an important resource of calanolides, which are dipyranocoumarins that inhibit the reverse transcriptase of human immunodeficiency virus type 1 (HIV-1 RT). Despite having great medicinal importance, enzymes involved in calanolide, biosynthesis and the pathway itself, are still largely unknown. Additionally, no genomic resources exist for this plant species.

**Results:**

In this work, we first analyzed the transcriptome of *C. brasiliense* leaves, stem, and roots using a RNA-seq strategy, which provided a dataset for functional gene mining. According to the structures of the calanolides, putative biosynthetic pathways were proposed. Finally, candidate unigenes in the transcriptome dataset, potentially involved in umbelliferone and calanolide (angular pyranocoumarin) biosynthetic pathways, were screened using mainly homology-based BLAST and phylogenetic analyses.

**Conclusions:**

The unigene dataset that was generated in this study provides an important resource for further molecular studies of *C. brasiliense*, especially for functional analysis of candidate genes involved in the biosynthetic pathways of linear and angular pyranocoumarins.

**Electronic supplementary material:**

The online version of this article (doi:10.1186/s12870-016-0862-9) contains supplementary material, which is available to authorized users.

## Background

*Calophyllum* sp. (Calophyllaceae) is a large group of tropical trees with more than 180–200 species [1]. Currently, some species of this genus have aroused great interest in the scientific community due to their promising phytochemical aspects. In Mexico, the most widely distributed species among the eight found in America is *Calophyllum brasiliense* Cambes [2], which grows in tropical rain forests from Brazil to northwest of Mexico [3]. This species contains a large number and variety of secondary metabolites including flavonoids, triterpenes, coumarins, chromones, and xanthones [4], some of which exhibit interesting anti-leishmanial, anti-bacterial, anti-cancer, anti-parasitic, and anti-viral properties [4, 5]. Two chemotypes have been classified according to their geographical origin. Chemotype 1 (CTP 1), which grows in Sierra de Santa Marta, State of Veracruz, Mexico, produces mammea type coumarins with high *in vitro* cytotoxic activity against human tumor cells and antibacterial properties against *Staphyloccoccus aureus*, *S. epidermidis* and *Bacillus subtilis* [6]. Meanwhile, chemotype 2 (CTP 2) grows in San Andres Tuxtla, State of Veracruz, Mexico, and produces calanolides, a series of tetracyclic dipyranocoumarins that exhibit an inhibitory effect against the reverse transcriptase of the human immunodeficiency virus type 1 (HIV-1 RT) [2, 7]. There are three different calanolides (A, B and C) that have been found in *C. brasiliense* and exhibit a significant inhibition on replication of the HIV-1 virus. Interestingly, these bioactive compounds show no toxicity to MT2 human lymphocytes [7]. Additional studies have shown that a high dose of B and C calanolides causes an increased number of spleen megakaryocytes and no alteration of hepatocytes [8]. Calanolide A, which possesses the highest inhibition of viral replication, has been synthesized and has been reported to have similar actions to the natural product [9, 10]. This molecule is in fact in clinical development as a novel therapeutic agent against HIV-1 infection [11, 12]. In plants, calanolides can be detected mainly in leaves (from CTP2), even if they come from seedlings of *C. brasiliense* that were germinated from seed and grown in a greenhouse [13]. Calanolides can be also detected in plant callus [13], cell suspension cultures, and leaves from 12-month-old plants that were *in vitro* regenerated from the young, nodal-stems of *C. brasiliense* plants [14].

The metabolic pathways in the biosynthesis of calanolides involve multiple and complex series of enzymatic reactions in which L-phenylalanine and *trans*-cinnamic acids can be considered as primary precursors. It is important to emphasize that some intermediates such as 7-hydroxycoumarin (umbelliferone) have interesting properties such as anti-fungal [15, 16] and insecticidal [17] activities, and they have also been shown to have strong inhibitory activity on proliferation of human bladder carcinoma E-J cell lines ([18] cited in [15]).

Despite the great pharmacological importance of *C. brasiliense,* the genomic basis of the synthesis and function of metabolic compounds such as calanolides remains poorly understood. Here, we present the first report of a complete transcriptome analysis of *C.brasiliense*. The main goal of this study was to characterize the transcriptome of *C.brasiliense* (CT2) for future gene identification and functional genomics studies of this species. We carried out *de novo* transcriptome sequencing and assembly of RNA libraries derived from terminal leaves, stems, and roots that come from *in vitro* regenerated *C. brasiliense* seedlings. We provide annotation to public databases and categorize the transcripts into biological functions and pathways. In addition, calanolide biosynthetic pathways are suggested, and based on the homologies of some genes, we propose some of them to be promising candidates for future analyses of the calanolide biosynthetic pathway.

## Results and discussions

### Sequencing and assembly

A total of 16,842,368 paired-end reads (2x150) were generated (5,276,841 for leaves, 5,000,558 for stem and 6,240,602 for roots). Prior to the assembly process, the paired reads were trimmed and/or merged together using the SeqPrep pipeline (see methods for more details). A *de novo* assembly was generated using Oases [19], a Bruijn graph-based assembler designed as an extension of Velvet [20] mainly used to assemble short-read sequences derived from transcriptomics data. Velvet/Oases produced a total of 61,620 contigs ranged from 0.1 to 10 kb, with an average length of 547.28 bp (Additional file 1). The GC contents of the contig set was approximately 44.7 %, which is similar to the GC content of the coding regions from other species within the Malpighiales order (reviewed in [21]). The N50 of these contigs was also estimated and resulted in a moderately high value of 867 bp. A fairly large number (40,727) of assembled contigs (40,727, which represents a 66.01 % of the total), were between 200 bp and 500 bp in length, indicating the presence of assembled fragments. For practical purposes, in the presented work, all contigs from the dataset are referred to as unigenes. The BLASTx algorithm [22] was used to annotate the unigenes based on the traditional top-BLAST-hit annotation method. As references, a collection of protein databases including the *Arabidopsis thaliana* (Arabidopsis) and plant RefSeq databases were used for this purpose. A significant value (e-value) of 10^−5^ was applied as threshold in the BLASTx similarity searches (Additional file 2: Table S1).

### Functional annotation

Based on the Arabidopsis top hits, Gene Ontology (GO) annotations for the *C. brasiliense* unigenes were obtained. WEGO software [23] was used to perform GO functional classification into the three major categories (biological process, molecular function, and cellular components). Among the unigenes with Arabidopsis hits, 42,090 (68.30 %) were assigned to different gene ontology categories with a total of 367,994 functional terms, of which 103,865 are unique. Biological processes comprised the majority of the functional terms (178,629; 48.54 %), followed by cellular components (95,428; 25.93 %) and molecular functions (93,937; 25.52 %) (Additional file 3: Figure S1; see also Additional file 4: Table S2). Top-ranked categories of GO biological processes were the sub-categories corresponding to cellular (27,090 unigenes) and metabolic (24,653 unigenes) processes. Interestingly, response to stimulus (14,101 unigenes) and biological regulation (12,646 unigenes) were also prominently represented among GO biological processes categories. In addition to functional annotation based on GOs, *C. brasiliense* unigenes were classified based on metabolic pathways available and described in Kyoto Encyclopedia of Genes and Genomes (KEGG). KEGG Automatic Annotation Server (KAAS; [24]) was used to assign to *C.brasiliense* unigenes the KEGG Orthology (KO) codes and enzyme commission (EC) numbers. KO codes were assigned to 4,881 unigenes, of which 1,733 could be associated to specific EC numbers related to 226 different metabolic pathways (Additional file 2: Table S1).

### Gene expression profiles of *C. brasiliense* organs

**Fig. 1:**
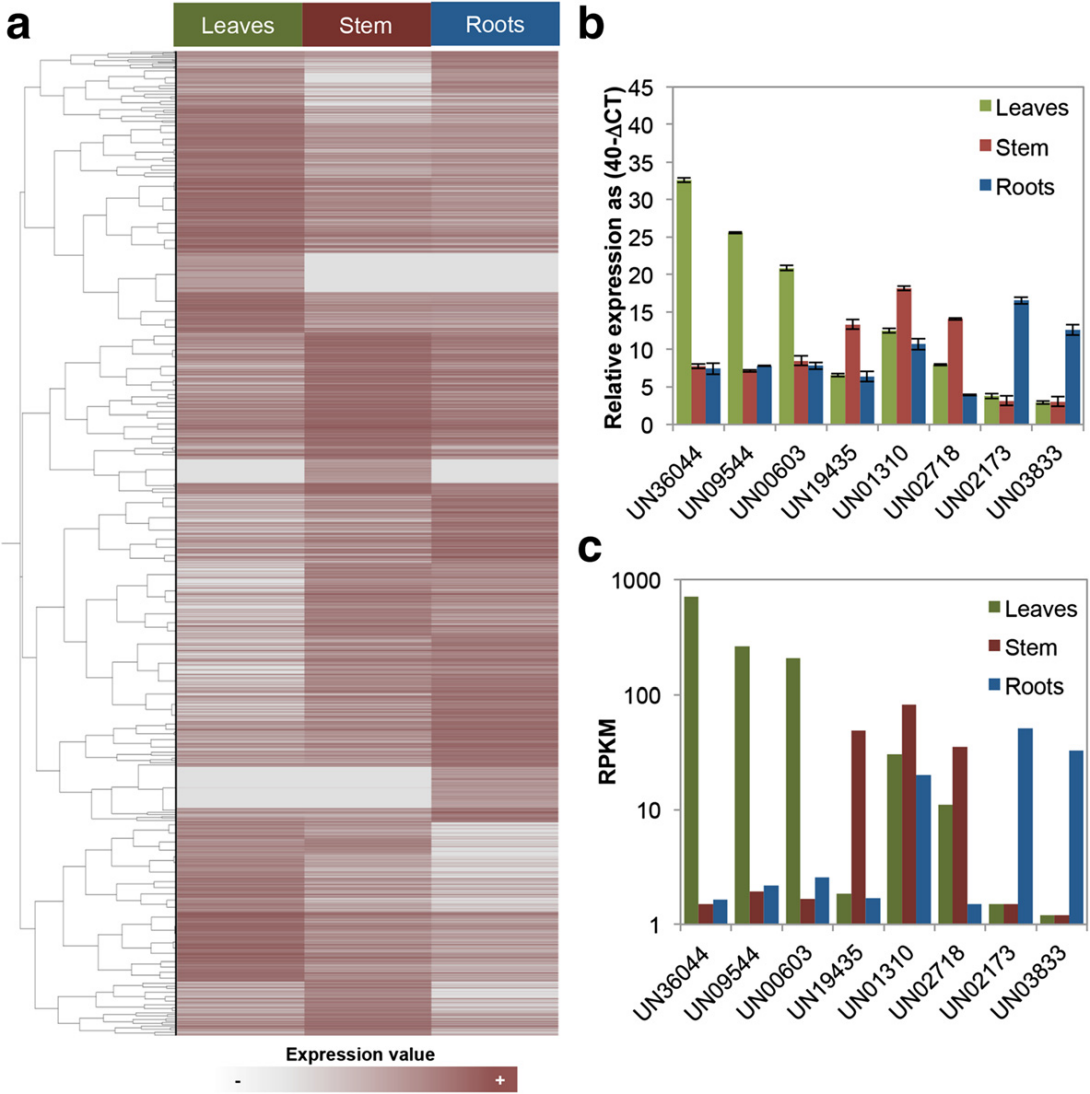
**a** Hierarchical clustering of *C. brasiliense* preferentially expressed unigenes based on RPKM values. The log_2_ RPKM values are presented with varying colors. The darker brown represents higher expression values, and the gray in the heat map lowest values. **b** RT-qPCR-based expression of eight-selected *C. brasiliense* preferentially expressed unigenes based on expression levels across organs (leaves, stem, and roots). Expression levels are given as 40-ΔCT, where ΔCT is the difference in threshold cycle number of the respective gene and the reference UN18770 (homolog to AT4G05050 which codify to UBQ11 on Arabidopsis); the number 40 was chosen because the PCR run stops after 40 cycles and a constant value was required as calibrator (see as example [76]). The results are shown as the averages ± SE of three biological replicate **c** RNA-Seq expression levels measured as reads per kb per million of reads (RPKM)

The gene expression profiles of roots, stems, and leaves were analyzed by mapping paired reads to the transcriptome assembly. An expression profile matrix containing the contigs (rows) and the number of mapped reads in each transcriptome sample (columns) was created (Additional file 4: Table S3). To make read counts comparable among samples, a normalization of reads per kilobase per million (RPKM) was performed. In order to distinguish expressed genes in at least one of the organs sampled from background, a threshold of RPKM ≥ 3 was used [25]. According to the stringency levels, 26,299 unigenes (59.26 % of the total) were expressed in all three tissues sampled (ubiquitous genes). The remaining unigenes were expressed in a single tissue (organ specifics) or they were distributed among different tissue combinations. The likelihood ratio statistic (R-statistic) [26] was calculated and used to identify those genes with significant expression level differences among sampled organs. Higher R-values indicate a greater probability of differential expression, whereas R-values near zero represent constitutive expression. In this context, it was considered as preferentially expressed genes the unigenes with *R*-values ≥ 8 (true positive rate of ~98 %) [26]. A total of 3,173 (7.15 %) *C. brasiliense* unigenes were identified as preferentially expressed genes, 1,274 in leaves, 965 in stems, and 934 in roots, some of which (247, 143 and 231, respectively) could be considered as organ-specific. (Fig. 1 and Additional file 4: Table S4).

In order to gain insight into the organ-function connection, the top 10 organ-specific unigenes were surveyed. In leaves, three *C. brasiliense* unigenes (UN36044, UN28345 and UN34582), which are homologous to Arabidopsis members of the glycine-aspartic acid-serine-leucine motif lipase/ hydrolase (GDSL lipase) family, were highly expressed as well as some homologs (UN09544 and UN13106) of 3-ketoacyl-CoA synthase proteins (KSC). Consistently, members of the GDSL lipase gene family, such as AT5G33370 and AT3G04290, are co-regulated with genes involved in cutin biosynthesis as CER6/KCS6/CUT1 (AT1G68530), which has a dominant role in the elongation of very-long-chain fatty acids for cuticular wax synthesis [27–29]. The lipoxygenase/peroxygenase pathway is also involved in biosynthesis of cutin monomers [30]; this could explain the expression profile of the UN00603, which was identified as a leaf-specific unigene and annotated as homologous to chloroplast lipoxygenase LOX2 (AT3G45140), an enzyme required for wound-induced jasmonic acid accumulation in Arabidopsis [31]. The presence of all of these genes in the leaf transcriptome was expected considering that epicuticular waxes are produced either exclusively during leaf development and expansion, or during the entire lifetime of the leaf [18].

Regarding stems, a homolog of fasciclin-like arabinogalactan protein FLA12 (AT5G60490; unigene UN35075) exhibited one of the highest expression levels as a stem-specific gene, which is consistent with previous reports showing that the expression of some members of the FLA gene family are correlated with the onset of secondary-wall cellulose synthesis in Arabidopsis stems, and with wood formation in the stems and branches of trees. This data suggests that unigene UN35075 may play a biological role in *C. brasiliense* stem development [32]. Additionally, genes encoding enzymes related to monolignol biosynthesis, such as phenylalanine ammonia-lyase (PAL1; UN01310), caffeoyl-CoA 3-*O*-methyltransferase (CCoAOMT; UN21637 and UN12250), and 4-coumarate: CoA ligase (4CL; UN01988), were identified as preferentially expressed in stems, although transcripts could be detected in all three organs sampled. Lignin, which plays a crucial role in conducting tissue in plant stems, is synthesized from the oxidative coupling of monolignols [33]. In addition, 16 unigenes homologous to Arabidopsis IRX proteins were also classified as specifically or preferentially expressed in stems; this was expected considering that the irregular xylem (*irx*) mutant is characterized by a reduction in cellulose in stem tissue [34]. Finally, two *C. barsiliense* unigenes (UN02173 and UN03226), which were homologous to members of the family of high affinity phosphorous transporters (PHT1), were identified as highly expressed only in roots, as well as the UN03833 unigene, a homolog of ARSK (AT2G26290), a poorly characterized gene encoding a root-specific kinase [35].

In order to validate the expression profiles obtained by normalized read counts, RT-qPCR was performed using nine chosen genes. All genes evaluated showed RT-qPCR expression profiles in complete agreement with the profiles derived from read counts analyses (Fig. 1).

### Functional annotation of preferentially expressed genes

**Fig. 2:**
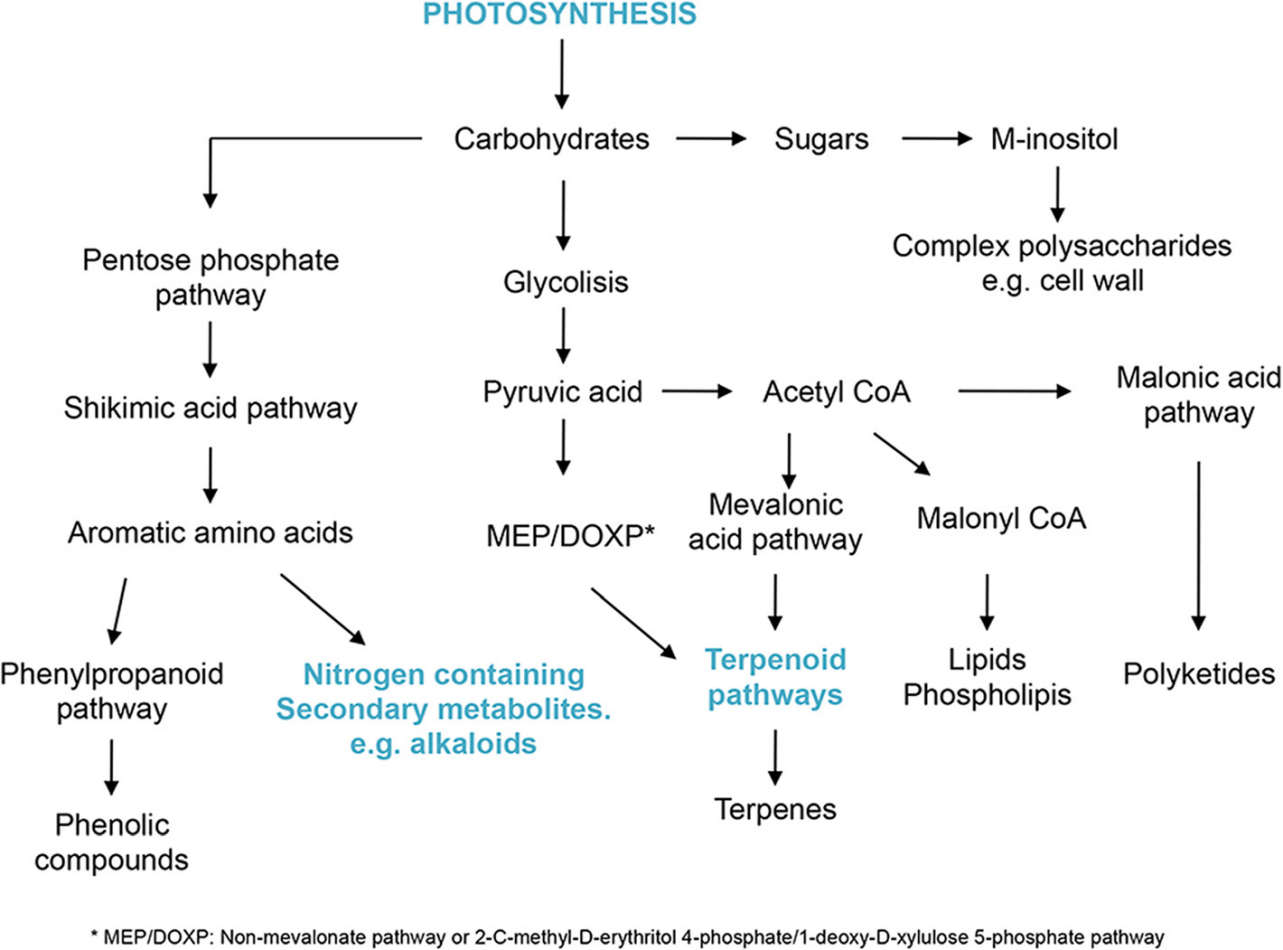
Principal biosynthetic pathways leading to synthesis of secondary metabolites (modified from [77]). Metabolic pathways shown in cyan represent the over-represented biological processes identified in leaves preferentially expressed genes

An enrichment analysis of GO terms was performed in order to identify, among the biological process categories, those subcategories that were over-represented in at least one of the organs analyzed (Additional file 4: Table S5). To compare the abundance of some preferentially expressed genes with specific biological processes, a modification of the same method applied to the preferentially expressed genes was used (the percentage of unigenes annotated with a given GO term in the organ-preferentially expressed genes was compared with the percentage of genes annotated with the same GO term in the complete set of the *C. brasiliense* transcriptome; Additional file 4: Table S6). Amoung the GO terms assigned to the sub-set of 3,173 of *C. brasiliense* preferentially expressed unigenes within biological process category, some over-represented biological processes were photosynthesis, alkaloid, and terpenoid metabolic-related (Additional file 3: Figure S2; see also Additional file 4: Table S7). This is consistent with the notion that the carbon skeletons of all secondary plant products are derived from carbohydrates synthesized by photosynthesis and that the synthesis of some of them depends on primary metabolites (Fig. 2). It should be noted that some biological processes showed no significant differences when the number of unigenes grouped for each process in each organ were compared. This was the case for the phenylpropanoid biosynthesis pathway (GO: 0009699), which showed an average of 39 genes that were grouped within this category in each organ sampled (Additional file 4: Table S6).

### The biosynthesis of umbelliferone, a key precursor in the calanolides formation

Coumarins are synthesized in plants via the shikimate pathway, in which phenylalanine is an end product that also gives rise to the aromatic amino acids tyrosine and tryptophan and other small molecules such as flavonoids and hydroxycinnamic acid conjugates [36]. Successive *para*- and *ortho*- hydroxylation of *trans*-cinnamate (conjugate base of *trans*-cinnamic acid) leads to the formation of coumarin via 2-coumarate, or via 4-coumarate, to the formation of hydroxycoumarins such as umbelliferone (7-hydroxycoumarin). Other hydroxycoumarins lacking oxygenation at C-7 also share the *trans*-cinnamic acid as its precursor. According to EC numbers assigned to *C. brasiliens*e unigenes, with only one exception (glutamate-prephenate aminotransferase; EC: 2.6.1.79), homologs from all enzymes required for the formation of L-phenylalanine via the shikimate pathway were identified (Additional file 3: Figure S3).

**Fig. 3:**
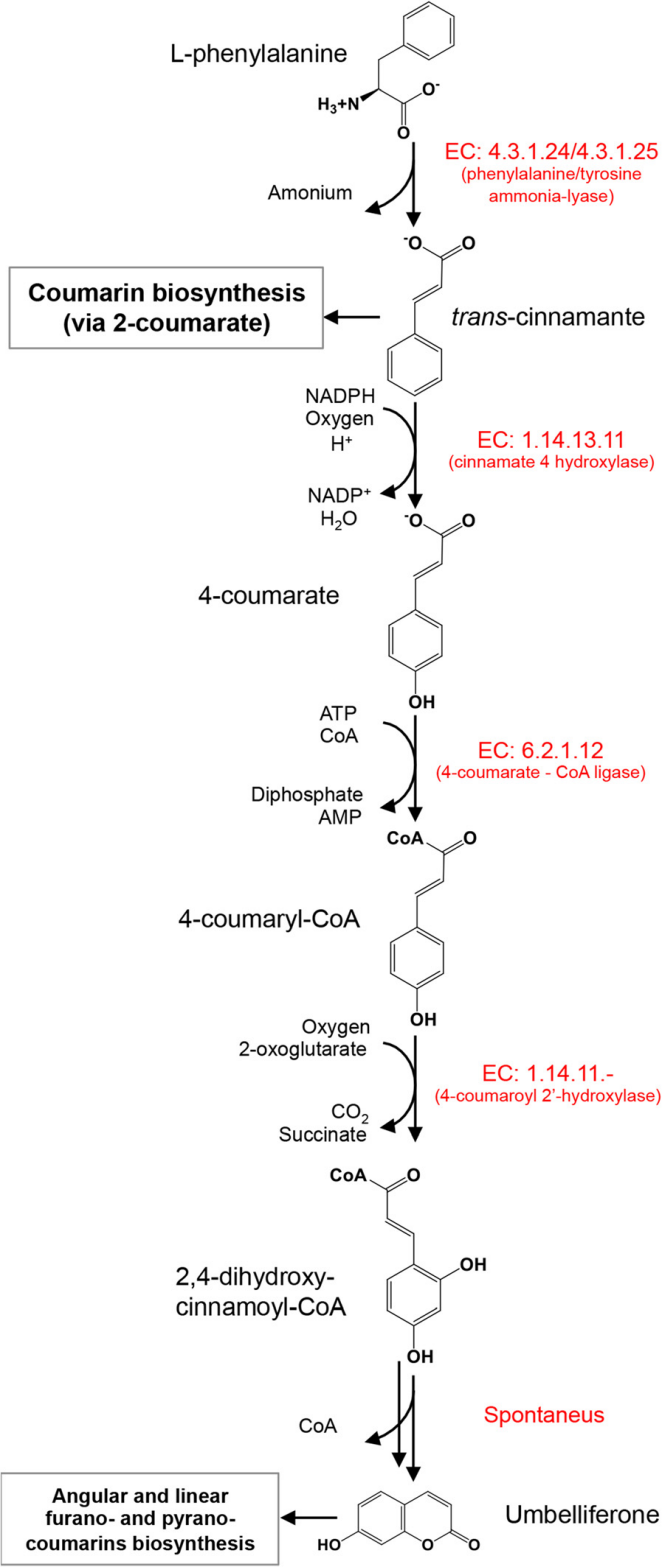
Biosynthetic pathway from umbelliferone starting with L-phenylalanine. The figure is an image taken and modified from the MetaCyc (http://metacyc.org/) search for umbelliferone biosynthesis

The key enzymes involved in the umbelliferone biosynthetic pathway (via 4-coumarate) are: a) phenylalanine ammonia lyase [PAL; EC:4.3.1.24], b) cinnamate 4 hydroxylase [C4H; EC:1.14.13.11], c) 4-coumarate: CoA ligase [4CL; EC:6.2.1.12], and d) 4-coumaroyl 2′-hydroxylase [EC:1.14.11.-] (Fig. 3). These enzymes involved in umbelliferone biosynthesis have been functionally characterized in *Ruta graveolens* species [39].

**Fig. 4:**
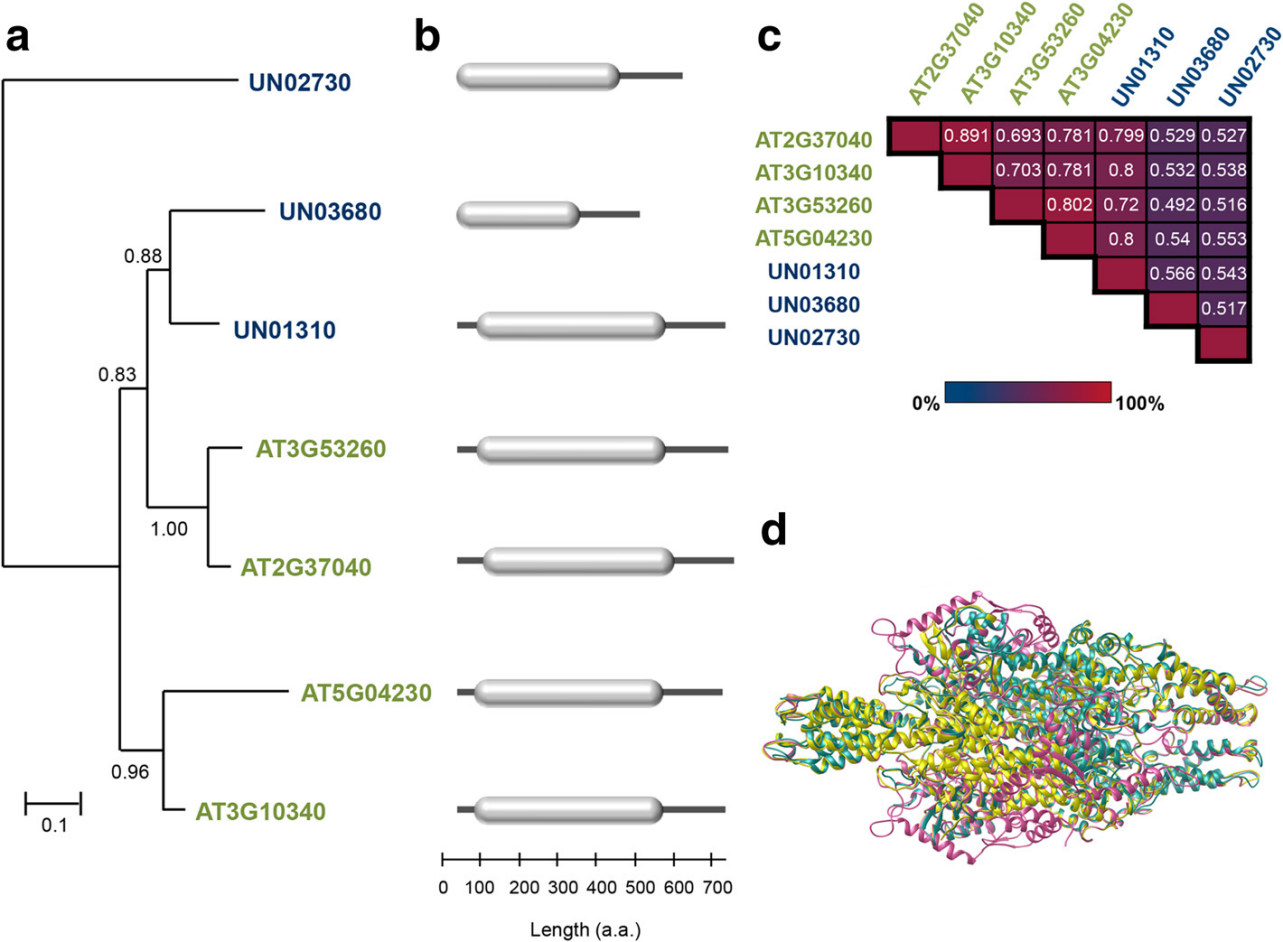
**a** Maximum likelihood phylogenetic tree depicting the relationship between the different *C. brasiliense* (blue) and Arabidopsis (green) PAL family members. Protein sequences were aligned with MUSCLE [69], and phylogenetic analyses were performed in maximum likelihood framework with SeaView v4.0 software [39]. **b** Primary protein structures in which the aromatic amino acid lyase conserved domain (PF00221) are represented by gray boxes. **c** Matrix of the percentage identities between the aligned protein sequences of PAL family members from *C. brasiliense* and Arabidopsis. **d** Structural model superposition of PAL proteins from *C. brasiliense* [UN01310 (pink), UN02730 (yellow) and UN03680 (cyan)]. The available structure of PAL protein from *Petroselinum crispum* in the Protein Database (PDB entry 1w27.1) was used as a template. The homology modeling was carried out using the SWISS-MODEL program and SWISS-PDB viewer v4.1.0 ([73, 75]; http://swissmodel.expasy.org/) was used to superpose the *C. brasiliense* PAL proteins

Proteins encoded by PAL gene family members can catalyze the conversion of L-phenylalanine to ammonia and *trans*-cinnamic acid, and are found in angiosperms and gymnosperms and also in some mosses, algae, fungi, and bacteria [37]. Based on the annotations extracted from KEGG, unigene UN01310 was identified as a homolog of PAL (K10775, EC:4.3.1.24). The WISE2 program [38] (http://www.ebi.ac.uk/Tools/psa/genewise/) and the Arabidopsis PAL1 protein (AT2G37040) were used to identify coding sequences in their correct open reading frames (this program uses a protein sequence as a template to predict a related protein sequence in the analyzed DNA sequence), then using the SeaView program [39], the protein-coding nucleotide sequences were aligned based on their corresponding amino acid translations to calculate the percent of identity at nucleotide and amino acid levels (Additional file 5). The identity between *C. brasiliense*/Arabidopsis *PAL* genes was estimated to be 69.05 % at the nucleotide level (CDS) and 79.9 % at the amino acid level. The distinctive aromatic amino acid lyase conserved domain (PF00221) was identified by motif/domain search against the Pfam database [40] (http://pfam.xfam.org/). Through a BLAST search based on the PF00221 domain, a total of eighteen *C. brasiliense* unigenes were identified as homologous to *PAL* genes, all of them annotated as homologs of some of the four existing Arabidopsis *PAL* genes ([41] PAL1; AT2G37040), PAL2; AT3G53260, PAL3; AT5G04230 and PAL4; AT3G10340) during the top-blast-hit annotation process (Additional file 2: Table S1). A phylogenetic tree from *C. brasiliense* PAL proteins was generated. Only the unigenes for which the proteins derived from their corresponding coding sequences represent at least 65 % of the length of their homologs were included (UN01310, UN03680 and UN02730). The *PAL* family members from Arabidopsis were also included in phylogenetic analysis. The multiple sequence alignments (Additional file 6) show that *C. brasiliense* proteins have high identity (ranged from 49.2 to 80.0 %) with PAL family proteins from Arabidopsis, and a three-dimensional structure analysis indicated that these proteins also contain a highly similar putative structure to other PAL plant proteins previously reported (Fig. 4). The high similarities of protein sequences among the *C. brasiliense* and Arabidopsis homologs and 3D models of the PAL family suggest that they may play similar functional roles.

Similar approaches to those described above were used to identify the remaining *C. brasiliense* genes involved in umbelliferone biosynthesis. A total of three unigenes were identified as homologous to the only *trans*-cinnamate 4-hydroxylase (AT2G30490; C4H) in the Arabidopsis genome (Additional file 3: Figure S4 and Additional file 7). C4H is a plant-specific cytochrome P450 (PF00067) that catalyzes the second step of the multibranched phenylpropanoid pathway [42]. Regarding 4-coumarate CoA ligase [4CL; EC:6.2.1.12], a total of six unigenes were detected as homologous to these proteins; however, only in three of them (UN01603, UN01988 and UN01725), were complete open reading frames identified (Additional file 8). The motif/domain searches revealed that both the AMP-binding C-terminal domain (PF13193) and common AMP-binding central domain (PF00501) are present in the translated sequences corresponding to these *C. brasiliense* unigenes (Additional file 3: Figure S5). Commonly, most angiosperms encode to a small family of 4CL (e.g., seven members in case of Arabidopsis). These enzymes are involved in the last step of the general phenylpropanoid pathway, and in addition to using 4-coumarate as substrate, they also convert p-coumaric acid, ferulic acid, caffeic acid and 5-OH-ferulic acid with different catalytic efficiency [43, 44].

Finally, the bi-directional best hit (BBH) method was used to identify a homolog of 4-coumaroyl 2′-hydroxylase [EC:1.14.11.-] from *Ruta graveolens*. The 4-coumaroyl 2′-hydroxylase isolated/characterized from *R. graveolens* (Accession JF799117.1) is the only enzyme that has been specifically assigned to coumarin synthesis, and to a lesser extent this enzyme also accepts 4-coumaroyl-CoA to produce umbelliferone [45]. The *C. brasiliense* unigene UN02124, in which the cytochrome P450 conserved domain PF00067 is present, showed 87.2 % identity with the published protein from *R. graveolens* (Additional file 3: Figure S6 and Additional file 9). The final step of the *in vivo* pathway for the synthesis of umbelliferone involves a *trans-cis* isomerization followed by a subsequent lactonization of the 2, 4-dihydroxy-cinnamoyl-CoA that closes the side chain, and this reaction occurs spontaneously (Fig. 3).

Considering that we were able to identify homologs to all genes involved in the umbelliferone biosynthetic pathway via 4-coummarate, and due the absence of several transcripts that potentially encode for enzymes such as cinnamate 2-hydroxylase (EC: 1.14.13.14), 2-coumarate *O*-β-glucosyltransferase (EC: 2.4.1.114), 2-coumarate β-D-glucoside isomerase (EC: 5.2.1.-) and coumarinic acid glucoside β-glucosidase (EC:3.2.1.21), which were first characterized in *Melilotus alba* [46, 47] were they are all involved in coumarin biosynthetic pathway via 2-coumarate, we suggest that in *C. brasiliense*, umbelliferone is synthesized via the 4-coumarate pathway. This was expected, because in contrast with mammals, only in a few plant species (e.g. *Catharanthus roseus* and *Conium maculatum*) has it been suggested that enzymes capable of carrying out the hydroxylation of coumarin in C-7 to produce umbelliferone exist (reviewed in [48]).

### Analysis of putative candidate genes involved in the calanolide (angular pyranocoumarins) biosynthetic pathway

**Fig. 5:**
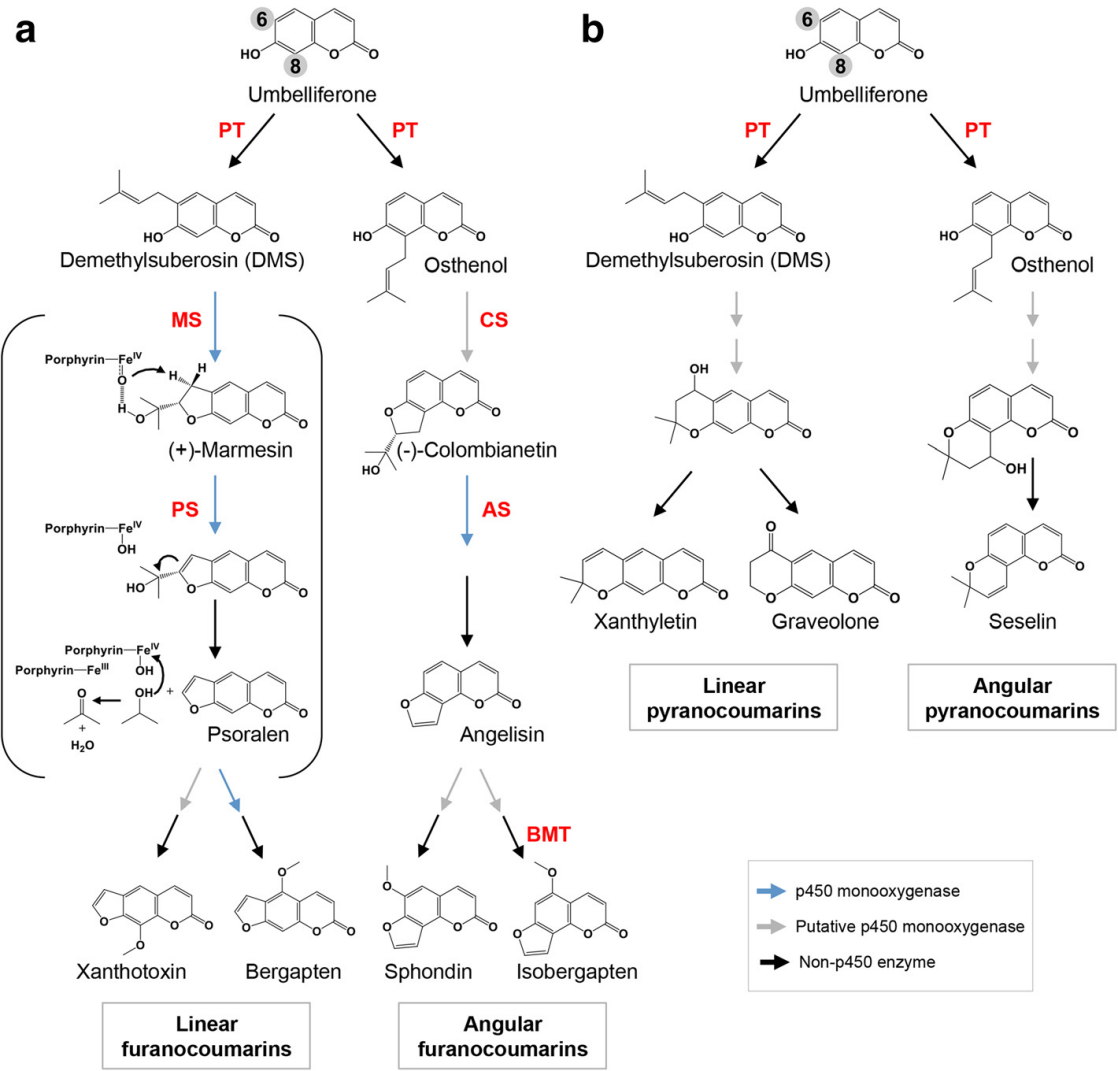
Biosynthetic pathways of both, linear and angular, furano- **a** and pyrano-coumarins **b**. Abbreviations of identified/characterized enzymes are shown in red: PS, umbelliferone prenyltransferase; MS, marmesin synthase; PS, psoralen synthase; CS, columbianetin synthase; AS, angelicin synthase; BMT, bergaptol *O*-methyltransferase. Details of the psoralen synthase-catalyzed reaction are in brackets. This figure was modified from [56]

The calanolides are pyranocoumarins, one class of coumarin derivates. The biosynthesis of pyranocoumarins is still poorly understood, but it has been reported that remarkable structural similarities between furanocoumarins and pyranocoumarins exist. Both classes of coumarins (furano- and pyrano- coumarins) can be grouped into linear and angular types. The determination of the type, either linear or angular, is based on the prenylation position of the common precursor (umbelliferone). In the case of the linear type, the pyran (or furan) ring is attached at C-6 and C-7, while in the angular type, the substitution is carried out at C-7 and C-8 [49]. Both types of pyranocoumarins are derived from the same precursors that give rise to linear and angular furanocoumarins (demethylsuberonosin and osthenol, respectively) (Fig. 5).

Previous studies conducted in *Pimpinella magna* and *Pastinaca sativa* plants could be considered the first experimental evidence to prove that linear and angular furanocuoumarins are derived from umbelliferone, prenylated at either the C-6 (leading to the formation of demethylsuberonosin; DMS) or C-8 position (osthenol), respectively [50]. In later years, additional investigations revealed that the cyclization of demethylsuberosin leads to (+)-marmesin formation, taking place through an enzymatic reaction that occurs in the presence of NADPH and molecular oxygen [51]. These ‘mamersin synthases’ have been identified as cytochrome P450 monooxygenases in *Petroselinum crispum* and *Ammi maju* plant species (reviewed in [52]). The range of reactions catalyzed by P450s include the epoxidation of olefins by insertion of an ‘oxen’ [53], and the reactive product of this reaction often inactivates the enzyme by alkylation of the prosthetic *heme* group [54]. However, no intermediate was released from mamersin synthase reaction, and it is likely that the 7-hydroxyl group of demethylsuberosin delocalizes the double bond electrons and favors the instantaneous cyclization to the dihydrofuranocoumarin (Fig. 5a). Model mechanisms have been proposed for the reactions mediated by catalytic action of the P450 enzymes, and one of these mechanisms consists of primary interaction of the catalytic P450 oxo-derivative, formed by heterolytic cleavage of the oxygen-oxygen bond in the ferric-hydroperoxy species, with aliphatic double bonds [52, 54]. This mechanism of reaction is compatible with such a cyclization of demethylsuberosin to (+)-marmesin, avoiding the formation of an intermediate epoxide (Fig. 5a). The formation of (−)-columbianetin from osthenol is catalyzed in an analogous fashion and the subsequent activities of both enzymes, angelicin and psoralen synthases, have been supported experimentally [55, 56]. It appears feasible that the synthesis of linear and angular pyranocoumarins such xanthyletin [57], graveolone [58], or seselin [59] may be produced concomitantly with furanocoumarins in a very similar way as from demethylsuberosin or osthenol, respectively (Fig. 5b).

**Fig. 6:**
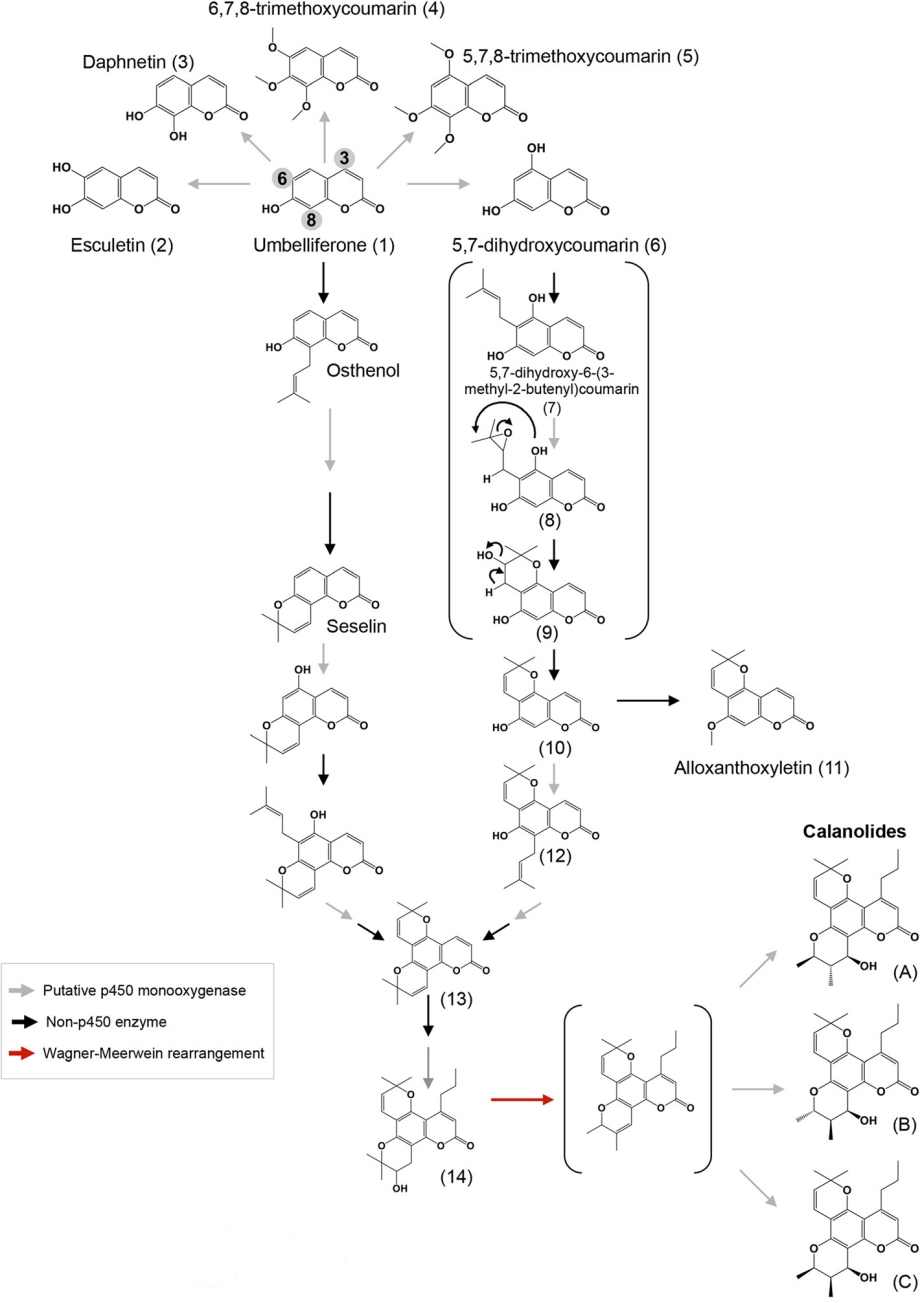
Proposed biosynthetic pathway for the calonolides **a**, **b** and **c** in *C. brasiliense*

Here, we propose two closely related biosynthetic pathways for calanolide biosynthesis (Fig. 6). These metabolic pathways imply some steps in the biosynthesis upon the basis of the presence of some related-compounds previously identified in other plant species. Starting from umbelliferone (1), a highly abundant metabolite identified in several plant species belonging to the Rutaceae and Compositae families [60], it is likely that the C-8 prenylated derivative (osthenol), which has been previously considered as a precursor at angular pyranocoumarins biosynthesis, could be one of the first intermediates. However, considering that some umbelliferone-related metabolites such as esculetin (2), daphnetin (3), 6,7,8-trimethoxycoumarin (4) and 5,7,8-trimethoxycoumarin (5), have been previously identified in several different plant species (reviewed in [49, 61]), it may be suggested that the formation of 5,7-dihydroxycoumarin (6) could be an initial step before the *c*-prenylation of umbelliferone at C-6, which leads to osthenol. Additional steps in calanolide biosinthesis include the epoxidation of 5,7-dihydroxy-6-(3-methyl-2-butenyl) coumarin (7) to (8), followed by the cyclization of (8) to (9), which after a dehydration step leads to (10). (10) could be a precursor of alloxanthoxyletin (11) [61], or in a similar way, a second *c*-prenylation at C-8 (12) may lead to (13) after their corresponding epoxidation-cyclization. Successive steps from (13) may involve the C-3 alkylation and the mono-hydroxylation of the pyran ring to produce (14), and then by Wagner-Meerwein rearrangement (1,2-methyl shifts), followed by the subsequent hydroxylation, the calanolides A, B and C could be produced (Fig. 6). Details from Wagner-Meerwein rearrangements that might lead to a calanolide formation (perhaps favoring the biosynthesis of one over the others) are shown in Additional file 3, Figure S7. The second suggested pathway could occur in a very similar way, starting with the *c*-prenylation of umbelliferone at C-8, which would lead to seselin production [59] and followed by a second *c*-prenylation at position C-4 (Fig. 6). Apparently, unlike the biosynthesis of furanocoumarins, in which the formation of an intermediary epoxide is avoided, the pyran ring formation in the pyranocoumarin biosynthetic pathway depends precisely on that intermediate.

Considering the remarkable structural similarities between furanocoumarins and pyranocoumarins (linear and angular), and the fact that both classes of compounds are derived from the same precursors (DMS and osthenol), it is possible to hypothesize that the enzymes involved in these biosynthetic pathways might share common ancestry. This hypothesis is consistent with the observation that furanocoumarins and pyranocuoumarins do not usually coexist in the same species. Even when many unknowns about biosynthesis and the function of furanocoumarins remain unresolved, it is clear that our knowledge about closely related compounds, such as pyranocoumarins, appears to be even less.

Concerning furanocoumarins, it has been reported that they are produced by a wide variety of plants in response to pathogen or herbivore attack. They are activated by ultraviolet light and can be highly toxic to certain vertebrate and invertebrate herbivores due to their integration into DNA, which contributes to rapid cell death [49, 62]. It has been suggested that linear and angular furanocoumarins are the results of a co-evolutionary process between plant and insects. Plant-insect interaction studies reveal that linear furanocoumarins are more toxic than angular ones; however, angular structures apparently produce a synergistic effect when they are combined with linear ones [62]. When mixed, linear and angular furanocoumarins result in a combination that is more difficult for insects to detoxify [62]. Apparently, during evolution, angular furanocoumarins appeared later than linear ones, a hypothesis that finds support based on the observation that angular furanocoumarins are always found concomitantly with linear structures, while linear types can be found alone (reviewed in [49, 63]).

During recent years, many enzymes involved in furanocoumarin biosynthesis have been described at the molecular level, including three P450 monooxygenases (psoralen-, angelicin-, and (+)-marmesin- synthases [55, 56]), bergaptol *O*-methyltransferase [64], as well as the key enzyme, a prenyltransferase [63]) involved in the critical step leading to precursors synthesis of linear and angular furanocoumarins (DMS and osthenol respectively; see Figs. 5 and 6). Additional P450-dependent enzymatic steps have remained unresolved, but the participation of this class of enzymes has been suggested in many steps of furanocoumarin biosynthesis. (Figs. 5 and 6, see [49] for an extended revision). Furanocoumarins are produced by a wide variety of plants in response to pathogen or herbivore attack.

The biosynthesis of pyranocoumarins has not yet been investigated. However, considering that psoralen synthase shows 70 % identity with angelisin synthase, and that its participation in linear and angular furanocoumarins biosynthesis has been previously demonstrated [55, 56], and based on the assumption that enzymes involved in pyranocoumarin biosynthesis might share a common ancestor of unknown functionality (perhaps as a result of gene duplications and subsequent molecular evolution), we used these and other enzymes involved in furanocoumarin biosynthesis as sequence references to identify homologs in the *C. brasiliense* unigenes set.

First, the prenyltransferase (PT) identified in parsley (*Petroselinum crispum*) [63], the angelicin synthase (CYP71AJ4) from *Pastinaca sativa*, the psoralen synthase (CYP71AJ1) identified from *Ammi majus* and their orthologs (CYP71AJ2 and CYP71AJ3 from *Apium graveolens* and *Pastinaca sativa*, respectively) [55, 56], were used as references in tBLASTn similarity searches (e-value 10^−6^) against the *C. brasiliense* unigene dataset. Additionally, the poorly characterized CYP82H1 isolated from *Ammi majus*, which increases its expression levels in plant-fungi interactions and accompanying furanocoumarin biosynthesis, was also included [65]. Sequences of unigenes that upon translation show at least 20 % identity over a resides window that represents a complete coding sequence (CDS), or at least 70 % of the homologous protein, were retained for future analysis.

A total of six PT-like sequences were identified. The subsequent comparison against PFAM databases confirmed the presence of the *Ubi*A prenyltransferase domain (PF01040). The unigene UN01964 (with 35 % shared identity) was resolved in the same clade as the parsley prenyltransferase (Additional file 3: Figure S8 and Additional file 10). Considering these results, we propose that UN01964 should be considered a leading candidate to encode a putative prenyl transferase involved in biosynthesis of the precursors that lead to synthesis of linear and angular pyranocoumarins.

Regarding the subsequent steps in pyranocoumarin biosynthesis, a total of 34 unigenes were identified as homologous to the previously characterized P450 monooxygeneases involved in the furanocoumarin biosynthetic pathway (stringency levels: coverage ≥ 70 %, identity ≥ 20 %). Three major clades were recognized on the phylogenetic tree, and one of these included only three *C. brasiliense* unigenes. The second clade brought together all CYP71AJ proteins that were included in phylogenetic analyses and a total of fourteen *C. brasiliense* unigenes. Finally, the third clade included the CYP82H1 P450 monooxygenase and the remaining *C. brasiliense* CYP-like sequences that were identified (Additional file 3: Figure S9, Additional file 11). The CYP71-related clade comprises two sister clades named as classes I and II, respectively. The translated Class I unigenes showed a percent identity that ranged from 31 to 46 % with respect to CYP71AJ proteins, while class II members were on average ~10 % less similar (Additional file 4: Table S8). Low percent similarities at the protein level were expected considering that the presence of furanocoumarins has not been reported in *C. brasiliense*, which is instead capable of producing pyranocoumarins (linear and angular, including the calonolide compounds) in young seedlings (mainly at leaves) and in callus cultures [13]. According to the expression profiles of these genes, 44 % were identified as preferentially expressed in some of the organs sampled. UN02363, UN03063, UN04124, and UN02841 were selected as preferentially expressed in leaves. With only one exception (UN02841), these genes were classified as CYP71-related; two of them (UN02363 and UN03063) grouped in class II and the other one into class I (UN04124). In addition, UN04124 possesses 35.6 and 35.8 % identity with the psoralen and angelicin synthases of *Pastinaca sativa*. Altogether, this data suggests that UN04124 can be considered a prime candidate for involvement in angular and/or linear pyranocoumarin biosynthesis.

## Conclusions

The unigene dataset generated in this study provides an important resource for further molecular studies of *C. brasiliense*, especially for characterizing candidate genes in the biosynthetic pathways of linear and angular pyranocoumarins. Using appropriate approaches, a series of candidate genes were identified. Consecutive analyses were conducted to determine their corresponding expression patterns and phylogenetic relationships. The candidate genes identified in *C. brasiliense* transcriptome that were suggested to possibly be involved in the biosynthesis of calanolides (angular coumarins), could be cloned and characterized in further studies. Additional bioinformatic analyses could be conducted in order to reduce the number of candidate proteins that may catalyze specific reactions at particular steps. We suggest that at least for P450 monooxygenase enzymes, docking and molecular dynamics analyses could be performed in order to reduce the number of candidate genes involved in the calanolide biosynthetic pathway.

## Methods

### Plan material

Previously, Bernabé et al., [13], have shown that callus cultures or young plants of *C. brasiliense* grown in a greenhouse are capable of producing calanolides. In order to guarantee the production of calanolides, *in vitro* seedlings were regenerated from the young nodal-stem of the same plants (chemotype 2) used previously by Bernabé et al. These plants were germinated from seeds collected in San Andrés Tuxtla, State of Veracruz. The plants were grown in a green house during 5–6 months approximately. Young nodal-stem were collected from the plants and used as the source of explants. Leaves, stems, and roots were collected from *in vitro* regenerated 12-month-old plants. According to the manufacturer’s instructions, total RNAs were isolated with TRIzol® Reagent (Life technologies). RNAs isolated from the three organs sampled, were re-purified with the RNeasy kit (Qiagen) and treated with RNase-free DNase I (Invitrogen) in order to remove the DNA residues. The quality and purity of RNAs were assessed with OD260/230 ratio by using the NanoDrop 2000 (Thermo Fisher). RNA integrity was evaluated by RNA integrity number (RIN) using an Agilent 2100 Bioanalyzer (Agilent Technologies). Only RNAs with RIN values ≥ 8.5 were used from library generation.

### cDNA library preparation and sequencing (RNA-seq)

cDNA preparation, library construction, and sequencing were performed at the Genomic Services laboratory, LANGEBIO-CINVESTAV, Mexico by using the MiSeq™ platform according to the manufacturer’s instructions (Illumina, San Diego, CA). Briefly, poly (A) RNA was isolated from 20 μg of total RNA using Sera-mag Magnetic Oligo (dT) Beads (Illumina). To avoid priming bias when synthesizing cDNA, the purified mRNA was first fragmented into small pieces. Then the double-stranded cDNA was synthesized using the SuperScript Double-Stranded cDNA Synthesis kit (Invitrogen, Camarillo, CA) with random hexamer (N6) primers (Illumina). The synthesized cDNA was subjected to end-repair and phosphorylation using T4 DNA polymerase, Klenow DNA polymerase and T4 PNK. These repaired cDNA fragments were 3′ adenylated using Klenow Exo- (3′ to 5′ exo minus, Illumina). Illumina paired-end adapters were ligated to the ends of these 3′-adenylated cDNA fragments, using specific barcodes for each sample (roots, stem, and leaves). Fifteen rounds of PCR amplification were performed to enrich the purified cDNA template using PCR Primer PE 1.0 and PE 2.0 (Illumina) with Phusion DNA Polymerase. The cDNA library was constructed with a fragment length range of 278 bp (±0.5 SD). Finally, after validating on an Agilent Technologies 2100 Bioanalyzer using the Agilent DNA 1000 chip kit, cDNA libraries were sequenced on a paired-end (2x150) flow cell using Illumina MiSeq sequencer. Files containing sequence reads and quality scores were deposited in the Short Read Archive of the National Center for Biotechnology Information (NCBI) [Accession number SRP079249].

### Data filtering and *de novo* assembly

Forward and reverse read pairs (generated by Illumina-MiSeq) were merged to form a single “longer-reads” using the SeqPrep pipeline (https://github.com/jstjohn/SeqPrep), with default parameters (a quality score cutoff of phred 33, a minimum merged read length of 15 bp and no mismatches in the overlapping region). Paired-end reads that did not overlap were trimmed with a sliding window approach (window size 10 bases, shift 1 base). The FASTX-Toolkit (http://hannonlab.cshl.edu/fastx_toolkit/index.html) was used to this purpose. Reads were discarded if they were smaller than 30 bases after trimming, orphan reads were also removed in order to keep pairs only. Velvet assembler using the Oases module [19] was used for sequence assembly. A unigenes set from *C. brasiliense* was generated considering only resulting contigs with a minimum size of 100 bp.

### Annotation of *C. brasiliense* unigenes

To annotate unigenes obtained by *de novo* assembly, we performed sequence similarity searches using the BLASTx algorithm (e-value 10^−5^) on *Arabidopsis thaliana* (TAIR v11), and other plant proteins (NCBI; ftp://ftp.ncbi.nlm.nih.gov/refseq/release/plant/) databases. Top protein matches from Arabidopsis or additional plant proteins were assigned to each of the *C. brasiliense* unigenes. The gene ontology (GO) functional classes and pathways for each *C. brasiliense* unigene were assigned based on Arabidopsis GO SLIM and pathway annotation (ftp://ftp.arabidopsis.org/home/tair/Ontologies/). The data were statistically analyzed using the WEGO software [23] which is a useful tool for plotting GO annotation results. Additionally, the unigenes were also analyzed using the KEGG Automatic Annotation Server (KAAS; http://www.genome.jp/tools/kaas/) to provide annotations of KEGG Orthology (KO) codes. The bi-directional best hit (BBH) method was used. Enzyme Commission (EC) numbers were also assigned based on the annotations extracted from Kyoto Encyclopedia of Genes and Genomes (KEGG) and were cross-checked with orthologous gene annotation projections from Plant Metabolic Network (PMN; http://www.plantcyc.org/), and if available, corresponding EC-formatted MetaCyc [66] cross-references were added.

### Expression profile analysis of *C. brasiliense* transcriptome

After assembling of the *C. brasiliense* transcriptome, every RNA-seq library was separately aligned to the generated transcriptome assembly using Bowtie [67]. Counting of alignments was done using RSEM [68]. Differential expression statistical analysis was done using the FPKM (fragments per Kilo bases of contigs for per million mapped reads) values and statistical method described by Sketel [26]. Briefly, all clusters were submitted to a log likelihood ratio statistics which tends asymptotically to a χ2 distribution as described by Stekel. It is based on a single statistical test to describe the extent to which a gene is differentially expressed between libraries. This method permits in any number of libraries to identify differential expressed genes.

### RT-qPCR

In order to identify the coding sequence in their correct open reading frame, eight of the unigenes of *C. brasiliense* identified as differentially expressed genes were aligned versus their Arabidopsis homologues. Using the SeaView program, protein-coding nucleotide sequences were aligned based on their corresponding amino acid translations (Additional file 12). Gene-specific primer pairs (Additional file 4: Table S9), were designed using the Primer3 v.0.4.0 web tool (http://bioinfo.ut.ee/primer3-0.4.0/primer3/) and then used in RT-qPCR assays.

A total of 10 ug of total RNA was reverse transcribed using SuperScript® III Reverse Transcriptase (Life Technologies) according to the manufacturer’s instructions. qPCR of selected genes was carried out through SYBR green chemistry (Applied Biosystem) on a real time thermal cycler (AB7500, Applied Biosystem). UBQ11 (UN18770) was used as an internal control. The thermal cycling program was set to 95 °C for 5 min, 40 cycles of 95 °C for 30 s, 60 °C for 30 s, and 72 °C for 1 min. All reactions were run in duplicates of three biological replicates.

### Phylogenetic analyses

A maximum likelihood framework was used in phylogenetic analyses, which were performed with SeaView v2.4 software [39]. The alignments and phylogenetic analysis were drived by SeaView using Muscle [69] and PhyML [70] programs, respectively. Topology, branch lengths and, equilibrium frequencies, were optimized and the PhyML option was used under LG (Le and Gascuel) model [71]. The starting tree was determined using BioNJ, and both nearest-neighbor interchange (NNI) and subtree pruning and regrafting (SPR) algorithms for tree searching were used. Branch robustness was analyzed by approximate likelihood-ratio test (aLRT) [72].

### Proteins modeling

The *C. brasiliense’s* 3D protein structures were modeled by the rigid body grouping method, using the Swiss-Model workspace (http://swissmodel.expasy.org/) [73, 74]. This server is used to align the target sequences and template structure available in the Protein Data Bank (PDB). Once the template has been selected the 3D structure of the target sequences can be modeled. Templates used for modeling were 1w27.1 for phenylalanine ammonia lyases (PAL) and 3a9u.1 for 4-coumarate: CoA ligases (4CL). Each model generated was checked for various parameters that include Z, GMQE (Global Model Quality Estimation) and QMEAN (Qualitative Model Energy ANalysis) scores to assess the accuracy of the model. The modeled *C. brasiliense* proteins were superimposed onto their corresponding homologues structures using the SWISS-PDB viewer v4.1.0 program [75].

## Additional files

Additional file 1:
*C. brasiliense* unigenes set. (TXT 68107 kb) Additional file 2: Table S1. Annotation of *C. brasiliense* unigenes. (XLSX 20582 kb) Additional file 3: Figures S1-S9. Figure S1: Gene ontology classification of *C. brasiliense* unigenes. Figure S2: Biological processes assigned to differentially expressed genes. Figure S3: Re-constructed *C. brasiliense* metabolic network from glucose to *trans*-cinnamate. Figure S4: Phylogenetic relationships, primary protein structures and identities percent of *trans*-cinnamate 4-hydroxylases proteins. Figure S5: Structural and phylogenetic analysis of 4-coumarate: CoA ligases (4CL) from *C. brasiliense*. Figure S6: Alignment of the 4-coumaroyl 2′-hydroxylases from *C. brasiliense*. Figure S7: Schematic representation of the Wagner-Meerwein rearrangement. Figure S8: *C. brasiliense* prenyltransferases. Figure S9: Phylogenetic tree of selected *C. brasiliense* cytochrome P450. (PPTX 1219 kb) Additional file 4: Tables S2-S9. Table S2: Functional classification of *C. brasiliense* unigenes set based on Arabidopsis GO annotation. Table S3: Expression profile matrix of *C. brasiliense* unigenes. Table S4: Preferentially expressed genes in the organs sampled (leaves, stem and roots) of *C. brasiliense*. Table S5: Functional classification of preferentially expressed genes. Table S6: Number of preferentially expressed genes associated to specific GO terms. Table S7: Over-represented biological processes in specific organs. Table S8: Matrix of the percentage identities between the aligned cytochrome P450. Table S9: Primers used in RT-qPCR assays. (XLSX 16223 kb) Additional file 5: Coding sequences alignment between PAL-like *C. brasiliense* unigenes and Arabidopsis PAL1 protein. (TXT 8 kb) Additional file 6: Coding sequences alignment between *C. brasiliense* and Arabidopsis PAL proteins. (TXT 16 kb) Additional file 7: Coding sequences alignment between C4H-like *C. brasiliense* and Arabidopsis C4H protein. (TXT 6 kb) Additional file 8: Coding sequences alignment between *C. brasiliense* and Arabidopsis 4CL proteins. (TXT 18 kb) Additional file 9: Coding sequences alignment between unigene UN02124 of *C. brasiliense* and *Ruta graveolens* CYP98A22 protein. (TXT 3 kb) Additional file 10: Coding sequences alignment between PT-like unigenes from *C. brasiliense* and *Petroselinum crispum* PT protein. (TXT 9 kb) Additional file 11: Coding sequences alignment between CYP-like unigenes from *C. brasiliense* and CYP proteins from different plant species involved in furanocoumarins biosynthesis. (TXT 74 kb) Additional file 12: Coding sequences alignment between those unigenes from *C. brasiliense* and their corresponding Arabidopsis homologs used in RT-qPCR assays. (TXT 29 kb)
