# Supplementary material for: Identification of candidate genes related to calanolide biosynthesis by transcriptome sequencing of Calophyllum brasiliense (Calophyllaceae)
Source: BMC Plant Biol. 2016 Aug 15;16:177. doi: 10.1186/s12870-016-0862-9 (PMC4986372; doi:10.1186/s12870-016-0862-9)
Supplement: Additional file 3: Figures S1-S9. — Figure S1: Gene ontology classification of C. brasiliense unigenes. Figure S2: Biological processes assigned to differentially expressed genes. Figure S3: Re-constructed C. brasiliense metabolic network from glucose to trans-cinnamate. Figure S4: Phylogenetic relationships, primary protein structures and identities percent of trans-cinnamate 4-hydroxylases proteins. Figure S5: Structural and phylogenetic analysis of 4-coumarate: CoA ligases (4CL) from C. brasiliense. Figure S6: Alignment of the 4-coumaroyl 2′-hydroxylases from C. brasiliense. Figure S7: Schematic representation of the Wagner-Meerwein rearrangement. Figure S8: C. brasiliense prenyltransferases. Figure S9: Phylogenetic tree of selected C. brasiliense cytochrome P450. (PPTX 1219 kb) [file 12870_2016_862_MOESM3_ESM.pptx]

## Slide 1
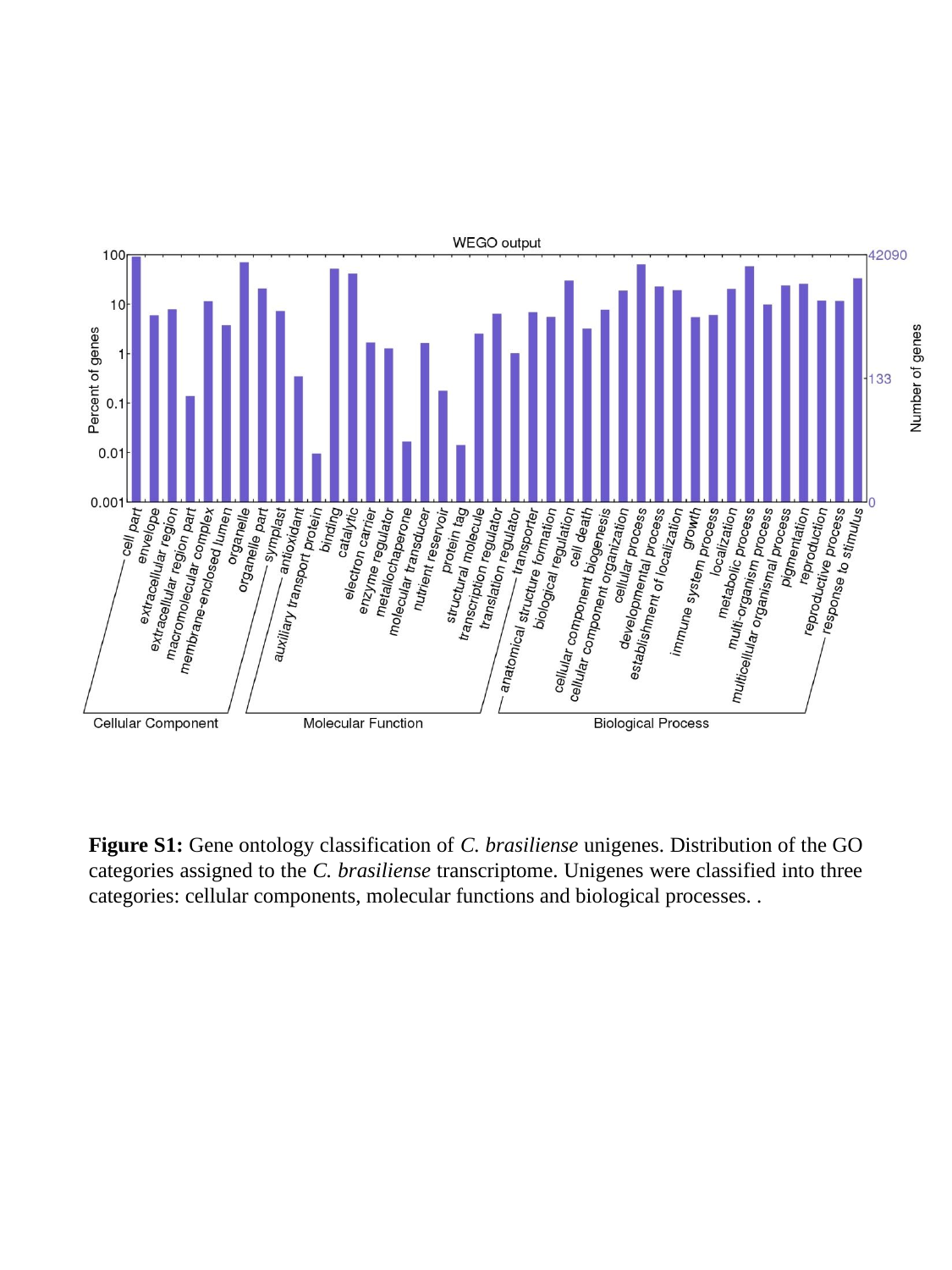

Figure S1: Gene ontology classification of C. brasiliense unigenes. Distribution of the GO categories assigned to the C. brasiliense transcriptome. Unigenes were classified into three categories: cellular components, molecular functions and biological processes. .

## Slide 2
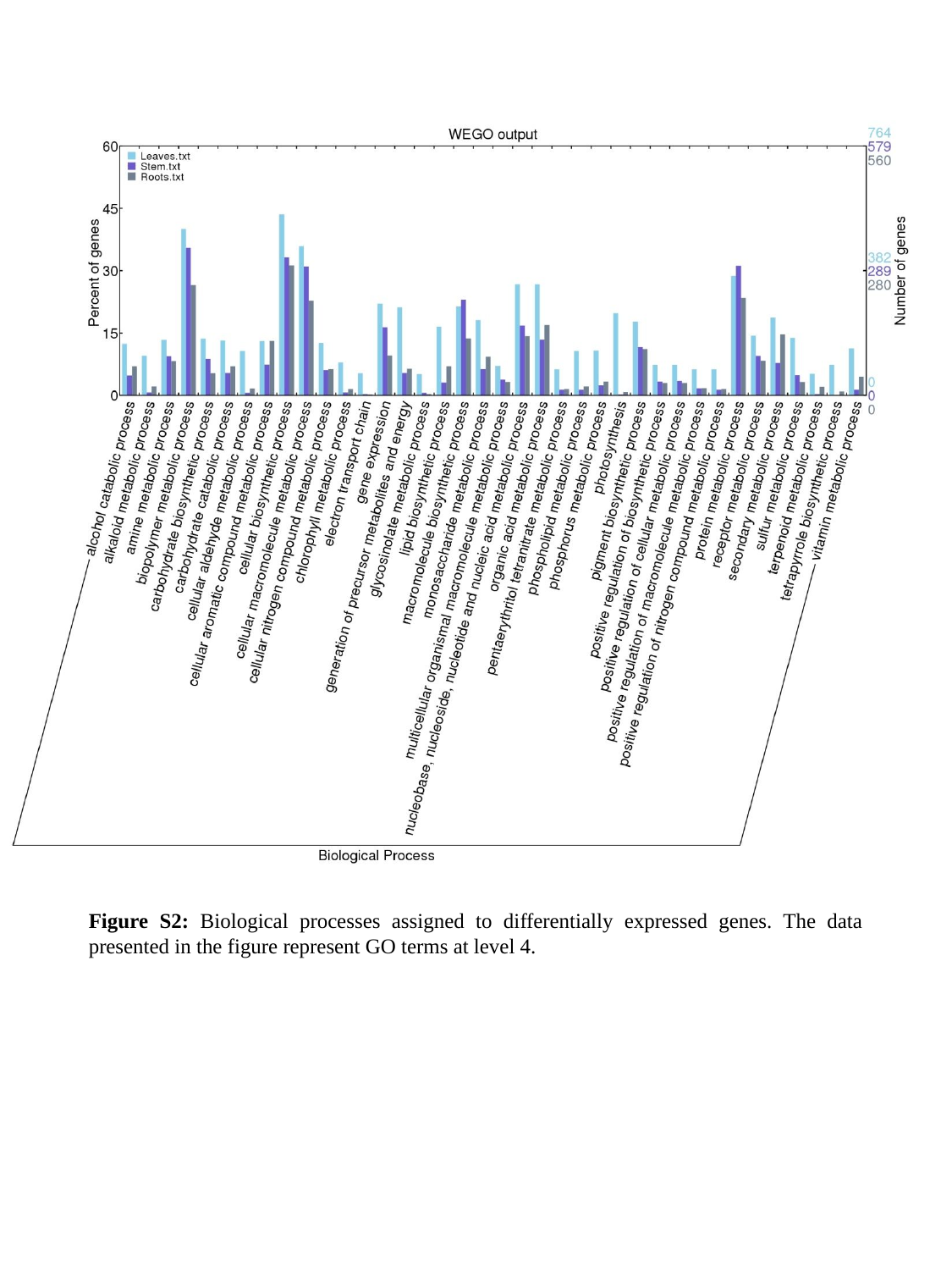

Figure S2: Biological processes assigned to differentially expressed genes. The data presented in the figure represent GO terms at level 4.

## Slide 3
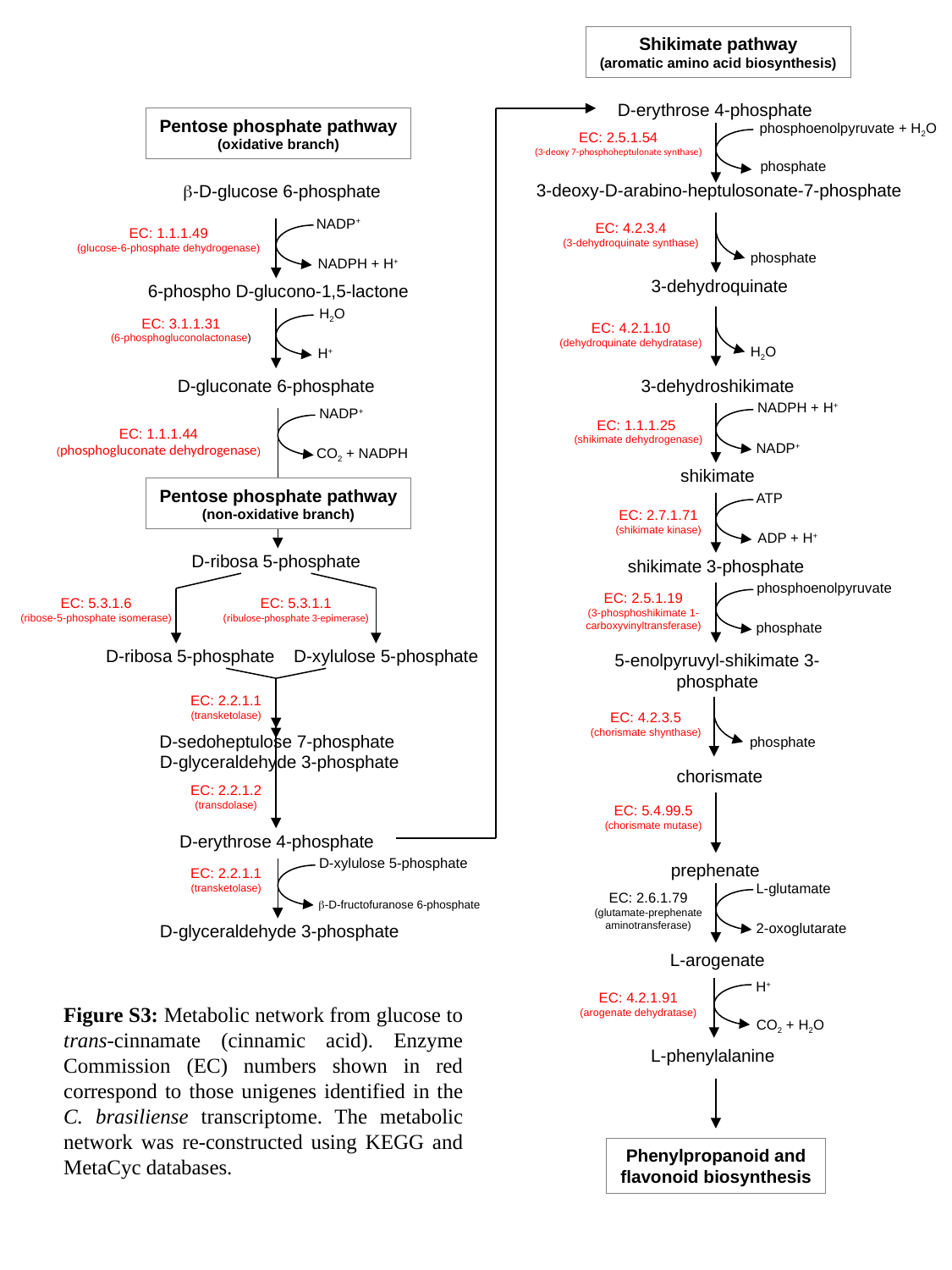

Shikimate pathway
(aromatic amino acid biosynthesis)
D-erythrose 4-phosphate
Pentose phosphate pathway (oxidative branch)
phosphoenolpyruvate + H2O
EC: 2.5.1.54
(3-deoxy 7-phosphoheptulonate synthase)
phosphate
3-deoxy-D-arabino-heptulosonate-7-phosphate
β-D-glucose 6-phosphate
NADP+
EC: 4.2.3.4
(3-dehydroquinate synthase)
EC: 1.1.1.49
(glucose-6-phosphate dehydrogenase)
phosphate
NADPH + H+
3-dehydroquinate
6-phospho D-glucono-1,5-lactone
H2O
EC: 3.1.1.31
(6-phosphogluconolactonase)
EC: 4.2.1.10
(dehydroquinate dehydratase)
H2O
H+
D-gluconate 6-phosphate
3-dehydroshikimate
NADPH + H+
NADP+
EC: 1.1.1.25
(shikimate dehydrogenase)
EC: 1.1.1.44
(phosphogluconate dehydrogenase)
NADP+
CO2 + NADPH
shikimate
Pentose phosphate pathway (non-oxidative branch)
ATP
EC: 2.7.1.71
(shikimate kinase)
ADP + H+
D-ribosa 5-phosphate
shikimate 3-phosphate
phosphoenolpyruvate
EC: 2.5.1.19
(3-phosphoshikimate 1-carboxyvinyltransferase)
EC: 5.3.1.6
(ribose-5-phosphate isomerase)
EC: 5.3.1.1
(ribulose-phosphate 3-epimerase)
phosphate
D-ribosa 5-phosphate
D-xylulose 5-phosphate
5-enolpyruvyl-shikimate 3-phosphate
EC: 2.2.1.1
(transketolase)
EC: 4.2.3.5
(chorismate shynthase)
D-sedoheptulose 7-phosphate
phosphate
D-glyceraldehyde 3-phosphate
chorismate
EC: 2.2.1.2
(transdolase)
EC: 5.4.99.5
(chorismate mutase)
D-erythrose 4-phosphate
D-xylulose 5-phosphate
prephenate
EC: 2.2.1.1
(transketolase)
L-glutamate
EC: 2.6.1.79
(glutamate-prephenate aminotransferase)
β-D-fructofuranose 6-phosphate
2-oxoglutarate
D-glyceraldehyde 3-phosphate
L-arogenate
H+
EC: 4.2.1.91
(arogenate dehydratase)
Figure S3: Metabolic network from glucose to trans-cinnamate (cinnamic acid). Enzyme Commission (EC) numbers shown in red correspond to those unigenes identified in the C. brasiliense transcriptome. The metabolic network was re-constructed using KEGG and MetaCyc databases.
CO2 + H2O
L-phenylalanine
Phenylpropanoid and flavonoid biosynthesis

## Slide 4
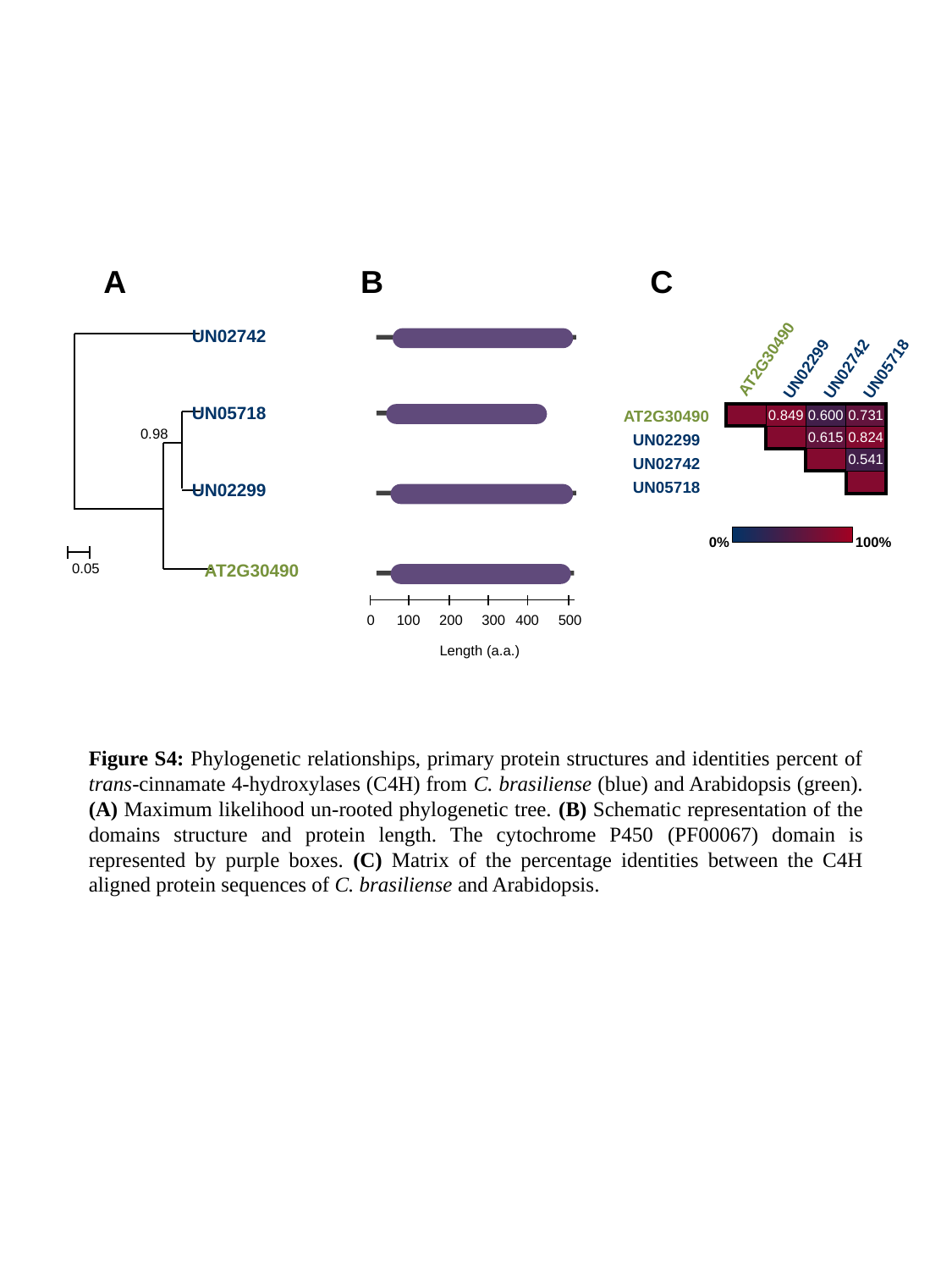

A
B
C
UN02742
UN05718
0.98
UN02299
AT2G30490
0.05
AT2G30490
UN02299
UN02742
UN05718
| AT2G30490 |
| --- |
| UN02299 |
| UN02742 |
| UN05718 |
| | 0.849 | 0.600 | 0.731 |
| --- | --- | --- | --- |
| | | 0.615 | 0.824 |
| | | | 0.541 |
| | | | |
0%
100%
100
200
300
400
500
0
Length (a.a.)
Figure S4: Phylogenetic relationships, primary protein structures and identities percent of trans-cinnamate 4-hydroxylases (C4H) from C. brasiliense (blue) and Arabidopsis (green). (A) Maximum likelihood un-rooted phylogenetic tree. (B) Schematic representation of the domains structure and protein length. The cytochrome P450 (PF00067) domain is represented by purple boxes. (C) Matrix of the percentage identities between the C4H aligned protein sequences of C. brasiliense and Arabidopsis.

## Slide 5
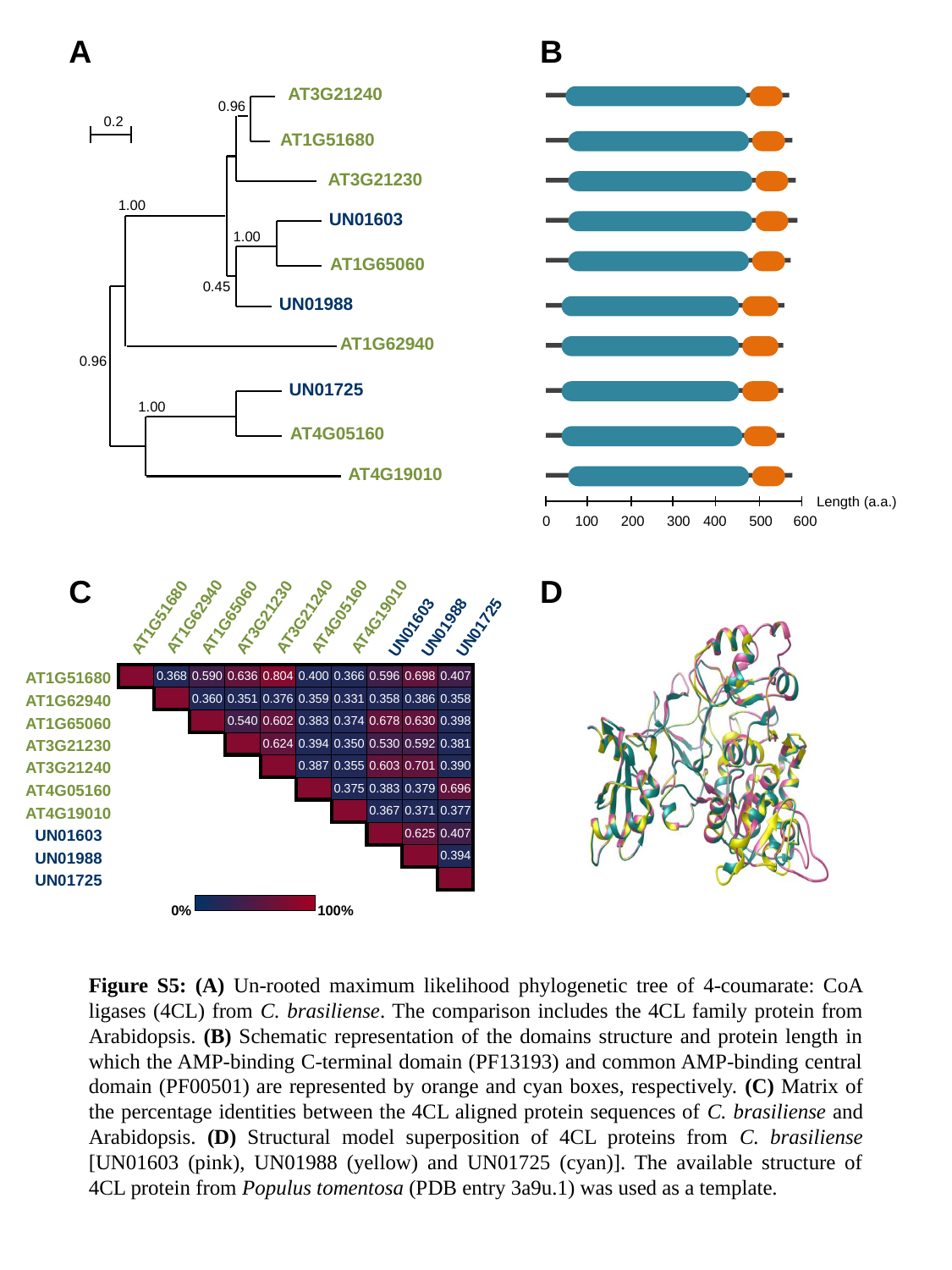

A
B
AT3G21240
0.96
0.2
AT1G51680
AT3G21230
1.00
UN01603
1.00
AT1G65060
0.45
UN01988
AT1G62940
0.96
UN01725
1.00
AT4G05160
AT4G19010
Length (a.a.)
100
200
300
400
500
600
0
C
D
AT1G62940
AT3G21240
AT4G05160
AT4G19010
AT1G51680
AT1G65060
AT3G21230
UN01603
UN01988
UN01725
| AT1G51680 |
| --- |
| AT1G62940 |
| AT1G65060 |
| AT3G21230 |
| AT3G21240 |
| AT4G05160 |
| AT4G19010 |
| UN01603 |
| UN01988 |
| UN01725 |
| | 0.368 | 0.590 | 0.636 | 0.804 | 0.400 | 0.366 | 0.596 | 0.698 | 0.407 |
| --- | --- | --- | --- | --- | --- | --- | --- | --- | --- |
| | | 0.360 | 0.351 | 0.376 | 0.359 | 0.331 | 0.358 | 0.386 | 0.358 |
| | | | 0.540 | 0.602 | 0.383 | 0.374 | 0.678 | 0.630 | 0.398 |
| | | | | 0.624 | 0.394 | 0.350 | 0.530 | 0.592 | 0.381 |
| | | | | | 0.387 | 0.355 | 0.603 | 0.701 | 0.390 |
| | | | | | | 0.375 | 0.383 | 0.379 | 0.696 |
| | | | | | | | 0.367 | 0.371 | 0.377 |
| | | | | | | | | 0.625 | 0.407 |
| | | | | | | | | | 0.394 |
| | | | | | | | | | |
0%
100%
Figure S5: (A) Un-rooted maximum likelihood phylogenetic tree of 4-coumarate: CoA ligases (4CL) from C. brasiliense. The comparison includes the 4CL family protein from Arabidopsis. (B) Schematic representation of the domains structure and protein length in which the AMP-binding C-terminal domain (PF13193) and common AMP-binding central domain (PF00501) are represented by orange and cyan boxes, respectively. (C) Matrix of the percentage identities between the 4CL aligned protein sequences of C. brasiliense and Arabidopsis. (D) Structural model superposition of 4CL proteins from C. brasiliense [UN01603 (pink), UN01988 (yellow) and UN01725 (cyan)]. The available structure of 4CL protein from Populus tomentosa (PDB entry 3a9u.1) was used as a template.

## Slide 6
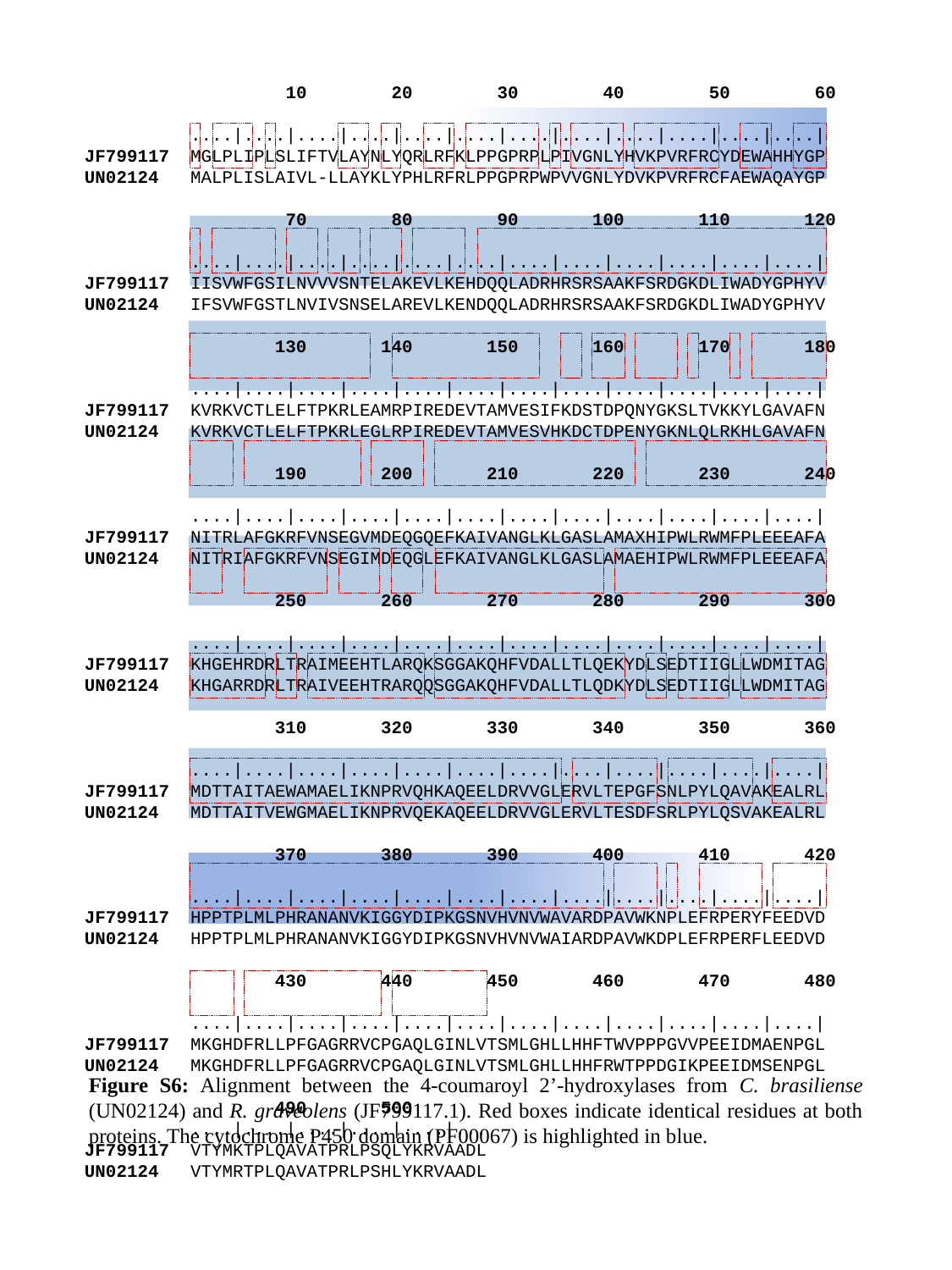

10 20 30 40 50 60
 ....|....|....|....|....|....|....|....|....|....|....|....|
JF799117 MGLPLIPLSLIFTVLAYNLYQRLRFKLPPGPRPLPIVGNLYHVKPVRFRCYDEWAHHYGP
UN02124 MALPLISLAIVL-LLAYKLYPHLRFRLPPGPRPWPVVGNLYDVKPVRFRCFAEWAQAYGP
 70 80 90 100 110 120
 ....|....|....|....|....|....|....|....|....|....|....|....|
JF799117 IISVWFGSILNVVVSNTELAKEVLKEHDQQLADRHRSRSAAKFSRDGKDLIWADYGPHYV
UN02124 IFSVWFGSTLNVIVSNSELAREVLKENDQQLADRHRSRSAAKFSRDGKDLIWADYGPHYV
 130 140 150 160 170 180
 ....|....|....|....|....|....|....|....|....|....|....|....|
JF799117 KVRKVCTLELFTPKRLEAMRPIREDEVTAMVESIFKDSTDPQNYGKSLTVKKYLGAVAFN
UN02124 KVRKVCTLELFTPKRLEGLRPIREDEVTAMVESVHKDCTDPENYGKNLQLRKHLGAVAFN
 190 200 210 220 230 240
 ....|....|....|....|....|....|....|....|....|....|....|....|
JF799117 NITRLAFGKRFVNSEGVMDEQGQEFKAIVANGLKLGASLAMAXHIPWLRWMFPLEEEAFA
UN02124 NITRIAFGKRFVNSEGIMDEQGLEFKAIVANGLKLGASLAMAEHIPWLRWMFPLEEEAFA
 250 260 270 280 290 300
 ....|....|....|....|....|....|....|....|....|....|....|....|
JF799117 KHGEHRDRLTRAIMEEHTLARQKSGGAKQHFVDALLTLQEKYDLSEDTIIGLLWDMITAG
UN02124 KHGARRDRLTRAIVEEHTRARQQSGGAKQHFVDALLTLQDKYDLSEDTIIGLLWDMITAG
 310 320 330 340 350 360
 ....|....|....|....|....|....|....|....|....|....|....|....|
JF799117 MDTTAITAEWAMAELIKNPRVQHKAQEELDRVVGLERVLTEPGFSNLPYLQAVAKEALRL
UN02124 MDTTAITVEWGMAELIKNPRVQEKAQEELDRVVGLERVLTESDFSRLPYLQSVAKEALRL
 370 380 390 400 410 420
 ....|....|....|....|....|....|....|....|....|....|....|....|
JF799117 HPPTPLMLPHRANANVKIGGYDIPKGSNVHVNVWAVARDPAVWKNPLEFRPERYFEEDVD
UN02124 HPPTPLMLPHRANANVKIGGYDIPKGSNVHVNVWAIARDPAVWKDPLEFRPERFLEEDVD
 430 440 450 460 470 480
 ....|....|....|....|....|....|....|....|....|....|....|....|
JF799117 MKGHDFRLLPFGAGRRVCPGAQLGINLVTSMLGHLLHHFTWVPPPGVVPEEIDMAENPGL
UN02124 MKGHDFRLLPFGAGRRVCPGAQLGINLVTSMLGHLLHHFRWTPPDGIKPEEIDMSENPGL
 490 500
 ....|....|....|....|....|...
JF799117 VTYMKTPLQAVATPRLPSQLYKRVAADL
UN02124 VTYMRTPLQAVATPRLPSHLYKRVAADL
Figure S6: Alignment between the 4-coumaroyl 2’-hydroxylases from C. brasiliense (UN02124) and R. graveolens (JF799117.1). Red boxes indicate identical residues at both proteins. The cytochrome P450 domain (PF00067) is highlighted in blue.

## Slide 7
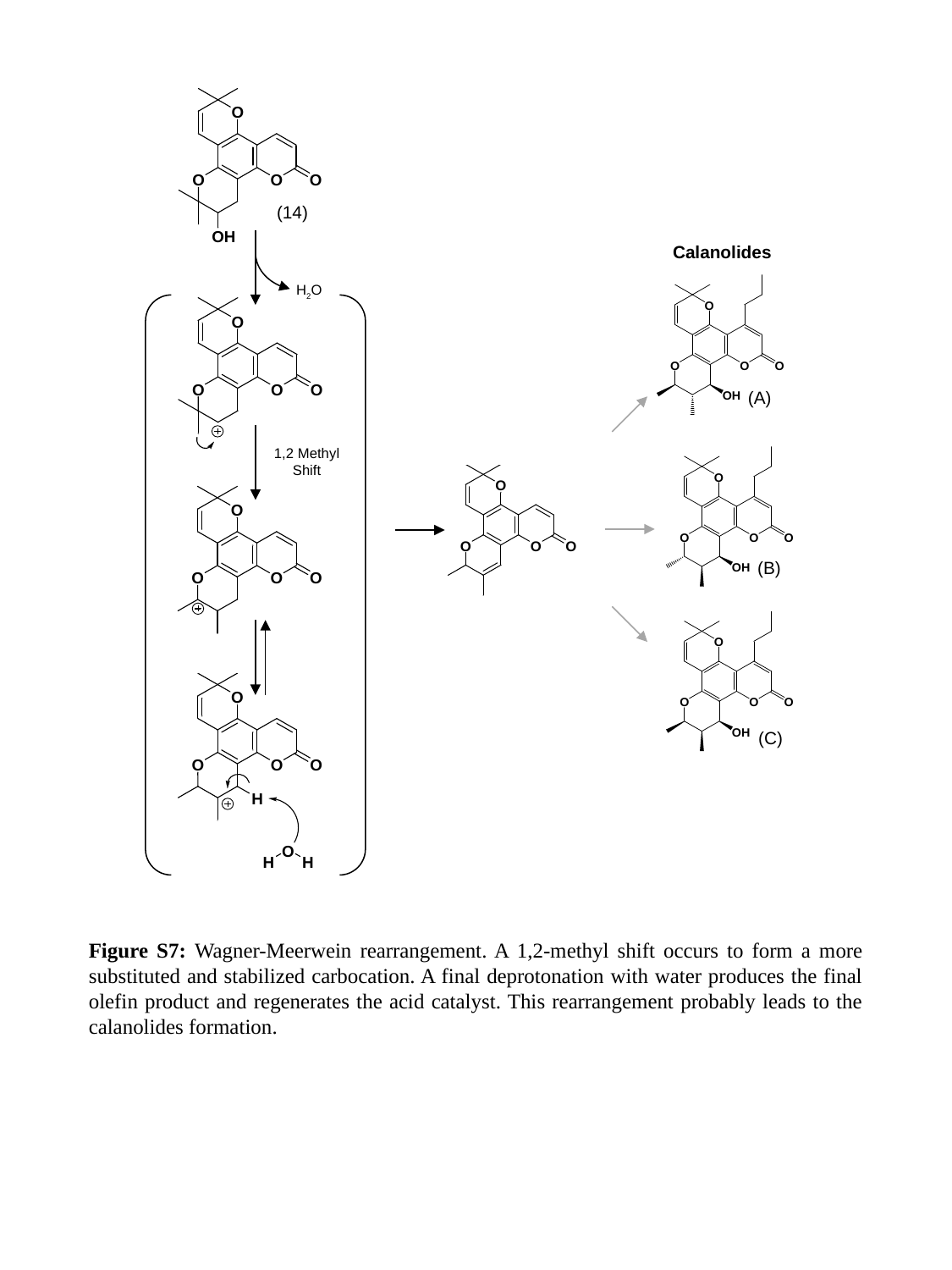

(14)
Calanolides
H2O
(A)
1,2 Methyl Shift
(B)
(C)
Figure S7: Wagner-Meerwein rearrangement. A 1,2-methyl shift occurs to form a more substituted and stabilized carbocation. A final deprotonation with water produces the final olefin product and regenerates the acid catalyst. This rearrangement probably leads to the calanolides formation.

## Slide 8
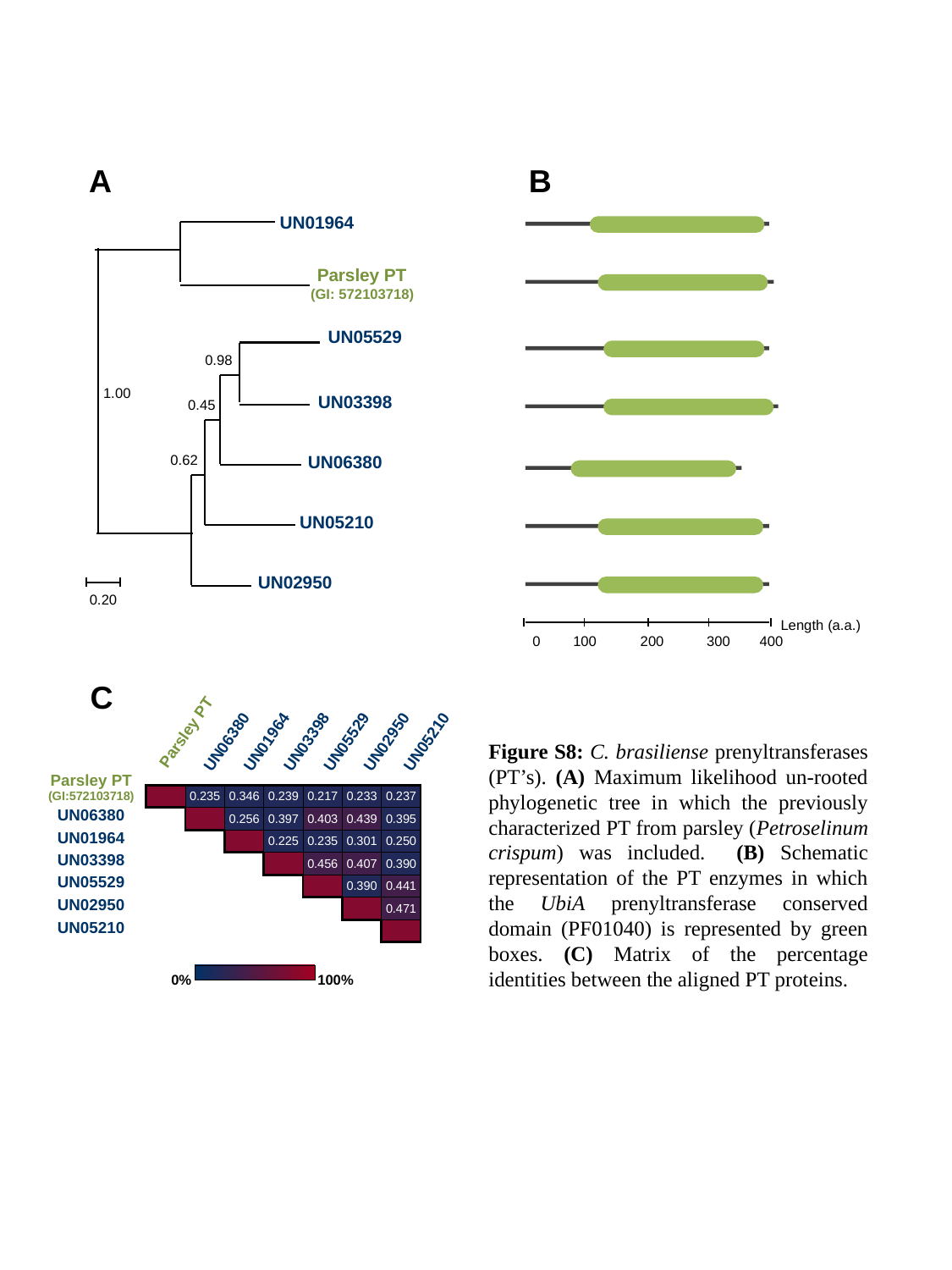

A
B
UN01964
Parsley PT
(GI: 572103718)
UN05529
0.98
1.00
UN03398
0.45
0.62
UN06380
UN05210
UN02950
0.20
Length (a.a.)
100
200
300
400
0
C
Parsley PT
UN06380
UN01964
UN03398
UN05529
UN02950
UN05210
Figure S8: C. brasiliense prenyltransferases (PT’s). (A) Maximum likelihood un-rooted phylogenetic tree in which the previously characterized PT from parsley (Petroselinum crispum) was included. (B) Schematic representation of the PT enzymes in which the UbiA prenyltransferase conserved domain (PF01040) is represented by green boxes. (C) Matrix of the percentage identities between the aligned PT proteins.
| Parsley PT (GI:572103718) |
| --- |
| UN06380 |
| UN01964 |
| UN03398 |
| UN05529 |
| UN02950 |
| UN05210 |
| |
| | 0.235 | 0.346 | 0.239 | 0.217 | 0.233 | 0.237 |
| --- | --- | --- | --- | --- | --- | --- |
| | | 0.256 | 0.397 | 0.403 | 0.439 | 0.395 |
| | | | 0.225 | 0.235 | 0.301 | 0.250 |
| | | | | 0.456 | 0.407 | 0.390 |
| | | | | | 0.390 | 0.441 |
| | | | | | | 0.471 |
| | | | | | | |
0%
100%

## Slide 9
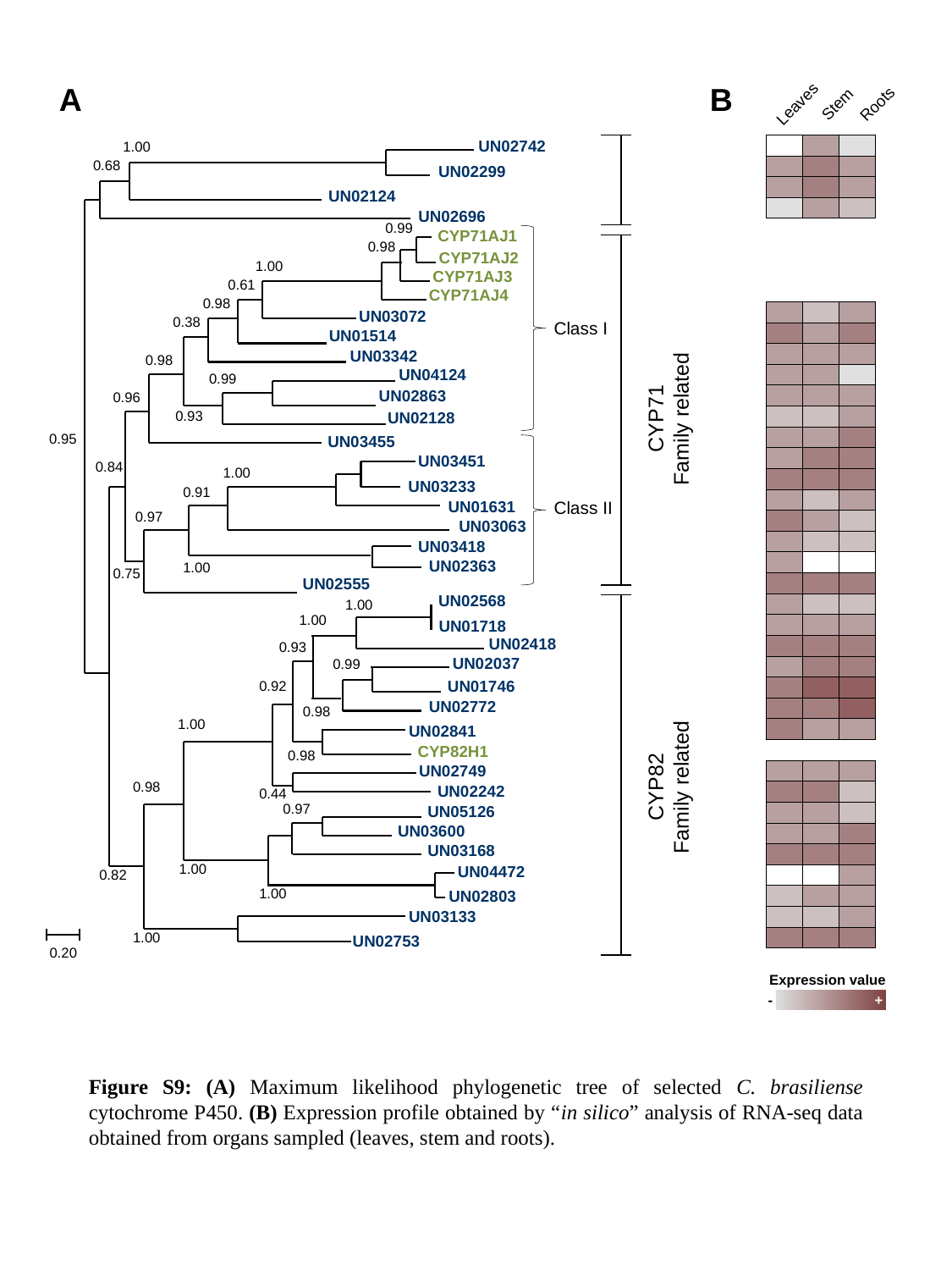

A
B
Leaves
Stem
Roots
UN02742
1.00
0.68
UN02299
UN02124
UN02696
0.99
CYP71AJ1
0.98
CYP71AJ2
1.00
CYP71AJ3
0.61
CYP71AJ4
0.98
UN03072
0.38
UN01514
UN03342
0.98
UN04124
0.99
UN02863
0.96
0.93
UN02128
0.95
UN03455
UN03451
0.84
1.00
UN03233
0.91
UN01631
0.97
UN03063
UN03418
UN02363
1.00
0.75
UN02555
UN02568
1.00
1.00
UN01718
UN02418
0.93
UN02037
0.99
UN01746
0.92
UN02772
0.98
1.00
UN02841
CYP82H1
0.98
UN02749
0.98
UN02242
0.44
0.97
UN05126
UN03600
UN03168
1.00
UN04472
0.82
1.00
UN02803
UN03133
1.00
UN02753
0.20
Class I
CYP71
Family related
Class II
CYP82
Family related
| | | |
| --- | --- | --- |
| | | |
| | | |
| | | |
| | | |
| | | |
| | | |
| | | |
| | | |
| | | |
| | | |
| | | |
| | | |
| | | |
| | | |
| | | |
| | | |
| | | |
| | | |
| | | |
| | | |
| | | |
| | | |
| | | |
| | | |
| | | |
| | | |
| | | |
| | | |
| | | |
| | | |
| | | |
| | | |
| | | |
| | | |
| | | |
| | | |
| | | |
| | | |
Expression value
-
+
Figure S9: (A) Maximum likelihood phylogenetic tree of selected C. brasiliense cytochrome P450. (B) Expression profile obtained by “in silico” analysis of RNA-seq data obtained from organs sampled (leaves, stem and roots).
